# Characterization of a new pathway that activates lumisterol *in vivo* to biologically active hydroxylumisterols

**DOI:** 10.1038/s41598-017-10202-7

**Published:** 2017-09-12

**Authors:** Andrzej T. Slominski, Tae-Kang Kim, Judith V. Hobrath, Zorica Janjetovic, Allen S. W. Oak, Arnold Postlethwaite, Zongtao Lin, Wei Li, Yukimasa Takeda, Anton M. Jetten, Robert C. Tuckey

**Affiliations:** 1 Department of Dermatology, VA Medical Center, Birmingham, AL 35249 USA; 2 Comprehensive Cancer Center, Cancer Chemoprevention Program, VA Medical Center, Birmingham, AL 35249 USA; 3 Nutrition Obesity Research Center, University of Alabama at Birmingham, VA Medical Center, Birmingham, AL 35249 USA; 4 Pathology and Laboratory Medicine Service, VA Medical Center, Birmingham, AL 35249 USA; 5 Drug Discovery Unit, College of Life Sciences, University of Dundee, Dundee, DD1 5EH United Kingdom; 6 Department of Medicine, Division of Rheumatology and Connective Tissue Diseases, University of Tennessee HSC, and Memphis VA Medical Center, Memphis, TN 38163 USA; 7 Department of Pharmaceutical Sciences, University of Tennessee HSC, Memphis, TN 38163 USA; 8 Cell Biology Section, National Institute of Environmental Health Sciences, National Institutes of Health, Research Triangle Park, Durham, NC 27709 USA; 9 School of Molecular Sciences, The University of Western Australia, Perth, WA Australia

## Abstract

Using LC/qTOF-MS we detected lumisterol, 20-hydroxylumisterol, 22-hydroxylumisterol, 24-hydroxylumisterol, 20,22-dihydroxylumisterol, pregnalumisterol, 17-hydroxypregnalumisterol and 17,20-dihydroxypregnalumisterol in human serum and epidermis, and the porcine adrenal gland. The hydroxylumisterols inhibited proliferation of human skin cells in a cell type-dependent fashion with predominant effects on epidermal keratinocytes. They also inhibited melanoma proliferation in both monolayer and soft agar. 20-Hydroxylumisterol stimulated the expression of several genes, including those associated with keratinocyte differentiation and antioxidative responses, while inhibiting the expression of others including *RORA* and *RORC*. Molecular modeling and studies on VDRE-transcriptional activity excludes action through the genomic site of the VDR. However, their favorable interactions with the A-pocket in conjunction with VDR translocation studies suggest they may act on this non-genomic VDR site. Inhibition of RORα and RORγ transactivation activities in a Tet-on CHO cell reporter system, RORα co-activator assays and inhibition of (RORE)-LUC reporter activity in skin cells, in conjunction with molecular modeling, identified RORα and RORγ as excellent receptor candidates for the hydroxylumisterols. Thus, we have discovered a new biologically relevant, lumisterogenic pathway, the metabolites of which display biological activity. This opens a new area of endocrine research on the effects of the hydroxylumisterols on different pathways in different cells and the mechanisms involved.

## Introduction

The epidermis, as the outer most layer of the skin, provides a protective barrier against water loss and environmental insults.^1,2^. The epidermis cooperates with the pigmentary system in these functions^3, 4^. Epidermal keratinocytes have the highest concentration of 7-dehydrocholesterol (7DHC), the final intermediate in cholesterol biosynthesis by the Kandutsch-Russel pathway, in the body. The B ring of 7DHC absorbs ultraviolet B radiation (UVB, λ = 280–320), resulting in the bond between C9 and C10 being broken producing previtamin D3 which then undergoes thermal isomerization to form vitamin D3 (D3). With a high dose of UVB, previtamin D3 undergoes photoisomerization to lumisterol3 (L3) and tachysterol3 (T3)^5^. These reactions are reversible and dependent on the temperature and UVB dose. T3 is the most photoreactive product and undergoes UVB-driven conversion to L3 via pre-D3, making L3 the major photoisomer generated with prolonged UVB exposure^6, 7^.

The current view is that D3 is the only important biological regulator derived from photolysis of 7DHC. After its activation to 1,25(OH)_2_D3, D3 not only regulates calcium homeostasis, but displays anticancer activities and also has important pleiotropic effects which include regulation of proliferation, differentiation, apoptosis, and immune and endocrine activities^5^ In contrast, it has been assumed that L3 affects neither calcium metabolism nor has any other significant biological activity. Its formation has been used to explain why UVB-induced cutaneous production of pre-D3 does not lead to systemic D3 intoxication with prolonged UVB exposure^5, 8, 9^.

Until recently, it was believed that vitamin D activation only involved the sequential hydroxylations at C25 and C1: D3 → 25(OH)D3 → 1,25(OH)_2_D3^7, 10, 11^. Surprisingly, the finding that CYP11A1 (the first enzyme of steroidogenesis^12^) can hydroxylate the D3 side chain at C17, C20, C22 and C23^13–16^ and the D2 side chain at C20, C17 and C24^17, 18^, has revealed new pathways of D activation. These pathways operate *in vivo* ^13, 19, 20^ with the major intermediates and products being detectable in human serum and epidermis^19^. The intermediates/products are biologically active^21, 22^, acting as partial agonists on the vitamin D receptor (VDR)^22, 23^ and as inverse agonists on retinoic acid orphan receptors (ROR)α and γ^24^. RORs are expressed in normal and pathological skin; therefore, binding of these novel secosteroids^19, 25^ to RORs is likely relevant to the regulation of biological functions in this organ^26, 27^.

In addition to adrenals, gonads and placenta, CYP11A1 is also found in the skin where its expression is stimulated by UVB^28, 29^. Another substrate for CYP11A1 is 7DHC which is metabolized similarly to cholesterol, with initial hydroxylations producing 22(OH)7DHC and 20,22(OH)_2_7DHC, and subsequent cleavage producing 7-dehydro-pregnenolone (7DHP), under both *in vitro* and *in vivo* conditions^28, 30, 31^. 7DHP can be further metabolized by steroidogenic enzymes to ∆7-steroids^30, 31^. In the skin these ∆7-steroids can potentially absorb UVB resulting in their conversion to secosteroids with a short side chain, pregnacalciferol (pD) and pregnalumisterol (pL)^32, 33^. It should be noted that chemically synthesized secosteroids with a short side chain show antiproliferative, anticancer and antifibrotic activities^33–39^.

Our surprising recent finding that purified and reconstituted CYP11A1 can hydroxylate L3, producing 20(OH)L3, 22(OH)L3, 20,22(OH)_2_L3 and pL^40^, has formed the basis for the current study on the production of L3 metabolites, *in vivo*, in the human body and for the testing of their phenotypic activity in skin cells, and for an effort to identify candidate receptors.

## Results and Discussion

### *In vivo* detection of lumisterol metabolites

#### Lumisterol (L3)

**Figure 1:**
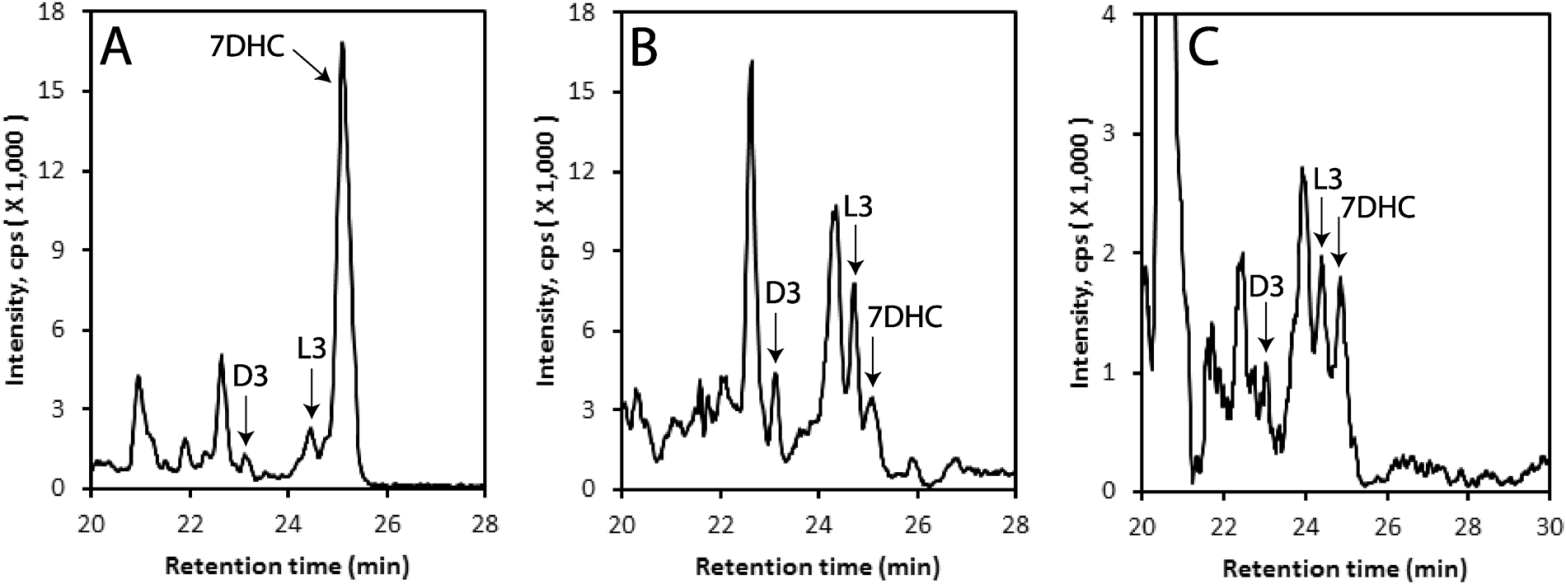
Detection of vitamin D3 (D3), lumisterol3 (L3) and 7-dehydrocholesterol (7DHC) in human epidermis (**A**), human serum (**B**) and pig adrenal grand (**C**). Extracted ion chromatograms (EIC) on qTOF LC-MS using *m/z* = 367.3 [M + H-H_2_O]^+^ are shown. The extraction and LC-MS conditions are described in Materials and Methods.

Analysis of extracts of human epidermis and serum, and pig adrenals, by LC/qTOF-MS alongside the corresponding standards, demonstrated the presence of 7DHC, D3 and L3 (Fig. 1). Epidermal samples were obtained from 13 patients including 6 African-Americans (AA) and 7 Caucasians (C), and sera were from a separate group of 13 individuals (12 C and 1 Hispanic). These were analyzed by LC/qTOF-MS to determine concentrations of 7DHC and L3 (Table 1). The concentration of 7DHC in human serum (~55 nM) is comparable to that reported for mouse serum^41^. In the epidermis, the level of 7DHC was 92-times higher than that of L3 but their concentrations in the serum were almost equal (Table 1). The serum concentration of L3 is 10-times higher than that previously reported for D3^19^ and the level in the epidermis is 13-times higher than that reported for D3^19^. The content of L3 and of its precursor 7DHC, in the epidermis or serum, showed no significant differences in relation to age, gender and race for these small sample sets (supplemental Figure 1). This is the first evidence that lumisterol formed in the skin can circulate in the serum and potentially accumulate in steroidogenic tissues such as the adrenal gland.

**Table 1:** Serum and tissue content of CYP11A1-derived metabolites of L3 in comparison to L3, 7DHC and D3 precursors.

	Serum (ng/ml)	Epidermis (ng/mg protein)
20(OH)L3	10.10 ± 1.85	8.34 ± 1.13
22(OH)L3	3.08 ± 0.77	12.19 ± 4.86
20,22(OH)_2_L3	1.25 ± 0.45	1.55 ± 0.96
pL	0.41 ± 0.24	0.06 ± 0.01
L3	19.19 ± 2.65	2.46 ± 1.13
D3	1.92 ± 0.34*	0.19 ± 0.05*
7DHC	21.28 ± 2.97	227.31 ± 59.38

The values represent means ± standard error (n = 13). *Values taken from ref. 19.

24(OH)L3 and hydroxyl-pLs were not quantitated because there were no peaks clear of background noise corresponding to their retention times under the conditions used.

#### Hydroxylumisterols

**Figure 2:**
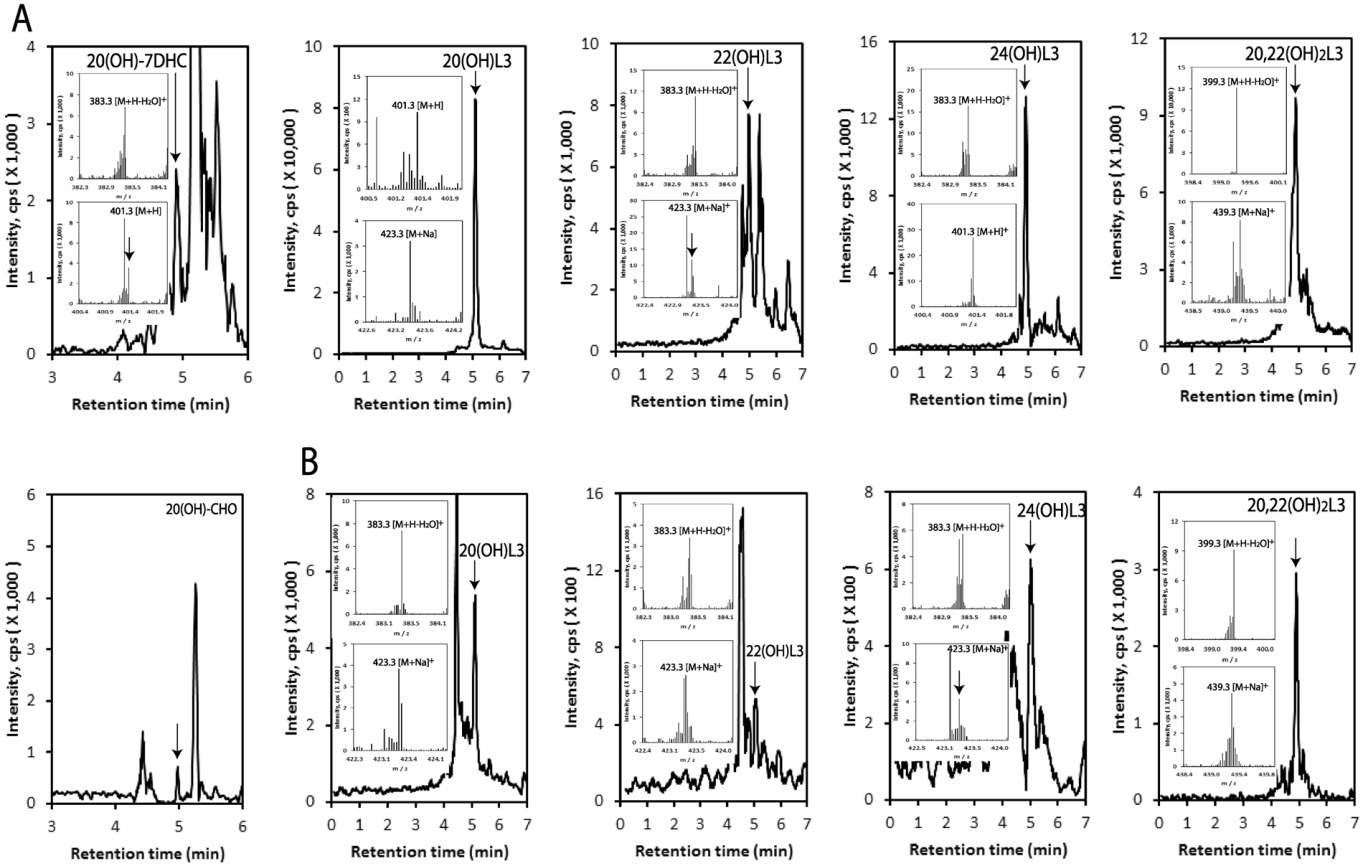
Detection of hydroxyl L3 derivatives in human epidermis (**A**) and human serum (**B**). Extracted ion chromatograms (EIC) on qTOF LC-MS are shown using *m/z* = 383.3 [M + H-H_2_O]^+^ for 20(OH)L3 (epidermis and serum), 22(OH)L3 (serum) and 24(OH)L3 (epidermis); 401.3 [M + H]^+^ for 22(OH)L3 (serum) and 24(OH)L3 (epidermis); 439.3 [M + Na]^+^ for 20,22(OH)_2_L3 (epidermis); 417.3 [M + H]^+^ for 20,22(OH)_2_L3 (serum). Inserts are mass spectra recorded on each indicated peak. The extraction and LC-MS conditions are described in Materials and Methods.

Lumisterol derivatives in extracts from the human epidermis and serum were first separated on a C18 column (25 cm long) with an acetonitrile in water gradient, as detailed in the materials and methods. The fractions with retention times (RT) corresponding to authentic standards of hydroxyderivatives of lumisterol or 20(OH)7DHC were then collected. The individual fractions were analyzed by UPLC on an Agilent Zorbax Eclipse Plus C18 column connected to a Xevo™ G2-S qTOF, with a methanol gradient as described by us previously^19^. Thus, identification of hydroxymetabolites that had identical masses was based on their RT compared to standards in two different solvent systems. From analysis of the extracted ion chromatograms (EIC) (see legend to Fig. 2 for monitored ions), we identified monohydroxy-metabolites of 7DHC and lumisterol with RT corresponding to chemically or enzymatically synthesized 20(OH)7DHC, 20(OH)L3, 22(OH)L3 and 24(OH)L3 standards (Fig. 2A). A dihydroxylumisterol was also identified with a RT corresponding to 20,22(OH)_2_L3 in the EIC of extracts of epidermis and serum (Fig. 2B). We also detected an ion at *m/z* = 367.3 (M + H-2H_2_O)^+^ with a RT corresponding to 20(OH)Chol in extracts of human epidermis and serum (Fig. 2). Thus, we have detected the known products of CYP11A1 action on lumisterol, namely 22(OH)L3, 24(OH)L3, and 20,22(OH)_2_L3 in human samples. These findings are also consistent with our previous *in vitro* studies that adrenal glands can transform L3 to 22(OH)L3, 24(OH)D3 and 20,22(OH)_2_L3^40^. 20(OH)L3 was not originally identified as a product of CYP11A1 action on lumisterol because insufficient material was available for NMR. Now, using chemically synthesized standard, we show that it corresponds to product G of CYP11A1 action on L3^40^. It has the same retention times as product G on a 25 cm C18 column with both methanol and acetonitrile solvent systems (supplemental Figure 2). This identification is consistent with the known ability of CYP11A1 to hydroxylate vitamin D3, 22 *R*(OH)cholesterol, and 22 *R*(OH)7DHC at this position^12, 14, 16, 28, 30, 31^. Furthermore, incubation of adrenal glands or HaCaT keratinocytes with exogenously-added L3 led to production of 20(OH)L3 (supplemental Figure 3).

Based on previous enzymatic studies^40^, the expression of CYP11A1 in the skin^28^ and our current data, we conclude that epidermal 20(OH)L3, 22(OH)L3, 24(OH)L3 and 20,22(OH)_2_L3 must be products of cutaneous CYP11A1-mediated metabolism of L3. Serum levels of these hydroxylumisterols may not only depend on their production rate in the skin, but also on their production rate from circulating L3 by the adrenal gland, the organ with the highest CYP11A1 concentration in the body^12^. Since the skin is intermittently exposed to UVB^42^, and the absorption of its energy by the unsaturated B ring of 7DHC^6–8^ or its hydroxyderivatives^43^ will ultimately lead to their transformation to compounds with the D3 or L3 configuration, UVB-induced transformation of locally produced 20(OH)7DHC, 22(OH)7DHC, or 20,22(OH)_2_7DHC^30, 31^ could represent an additional source of the detected hydroxylumisterol compounds.

#### Pregna-lumisterol (pL) and its hydroxyl-pL metabolites

**Figure 3:**
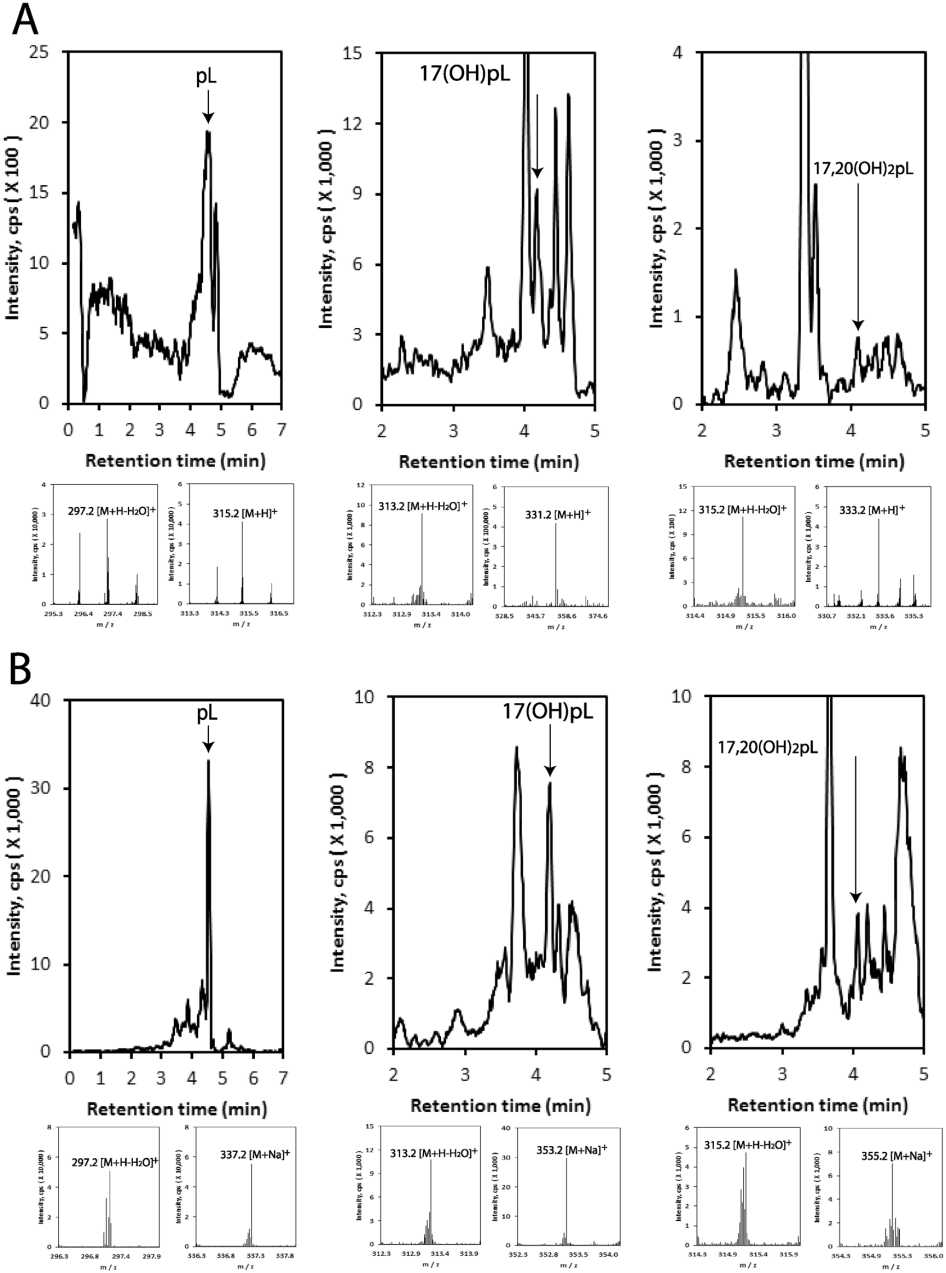
Detection of hydroxyl pL derivatives in human epidermis (**A**) and human serum (**B**). Extracted ion chromatograms (EIC) on qTOF LC-MS are shown using *m/z* = 297.2 [M + H-H_2_O]^+^ for pL (epidermis and serum); 313.2 [M + H-H_2_O]^+^ for 17(OH)pL (epidermis and serum); 333.2 [M + H]^+^ for 17,20(OH)_2_pL (epidermis); 315.2 [M + H-H_2_O]^+^ for 17,20(OH)_2_pL (serum). Mass spectra detected in each samples are shown below EICs. The extraction and LC-MS conditions are described in Materials and Methods.

We recently reported that pL is produced from L3 by purified CYP11A1 and fragments of adrenals, by cleavage of the lumisterol side chain^40^. Analysis of extracts of human epidermis and serum, and pig adrenals showed species corresponding to the retention times of standards of pL, 17(OH)pL, and 17,20(OH)_2_pL in the epidermis and human serum by UPLC/MS (using *m/z* = 297.2 [M + H-H_2_O]^+^, 315.2 [M + H]^+^ and 337.2 [M + Na]^+^ for pL; or of *m/z* = 313.2 [M + H-H_2_O]^+^, 331.2 [M + H]^+^ and 353.2 [M + Na]^+^ for 17(OH)pL; or of *m/z* = 315.2 [M + H-H_2_O]^+^, 331.2 [M + H]^+^ and 355.2 [M + Na]^+^ for 17,20(OH)_2_pL) (Fig. 3). 17(OH)pL and 17,20(OH)_2_pL were also detected in adrenal extracts with 17(OH)pL levels being increased by the addition of exogenous L3 (supplemental Fig. 4), indicating that pL is metabolized by steroidogenic enzymes within the adrenal. Epidermal production of pL and its subsequent hydroxylation is consistent with the steroidogenic activity of the skin, as discussed recently^44^. An additional source of pL, 17(OH)pL and 17,20(OH)_2_pL in the epidermis could be the UVB-induced photochemical transformation of 7DHP, 17(OH)7DHP, or 17,20(OH)_2_7DHC, respectively^28, 32, 34^, since these 5,7-dienes can be produced in the skin^30, 31^.

#### Tissue and serum concentrations of L3 metabolites

In the epidermis, the CYP11A1-derived mono-hydroxylumis-terols were present at significantly higher (p < 0.01) levels than the parental L3; however, the reverse was observed in serum (Table 1). The 22(OH)L3 concentration was comparable to the 20(OH)L3 level in epidermis but lower than 20(OH)L3 in serum, possibly reflecting different rates of clearance. The concentration of 20,22(OH)_2_L3 was significantly lower (p < 0.01) than either 22(OH)L3 or 20(OH)L3. pL showed the lowest concentration of the CYP11A1-derived metabolites analyzed in both epidermis and serum, consistent with it being only a minor product of CYP11A1 action on L3^40^. Full statistical analysis is provided in supplemental Table 1. Analyses of levels of L3 metabolites for gender, age and racial group showed no statistical difference in epidermal or serum concentrations (not shown). The serum concentrations of 20(OH)L3 is 9 times higher than that previously reported for 20(OH)D3 while the 22(OH)L3 concentration is similar to that reported for 22(OH)D3^19^. Epidermal levels of 20(OH)L3 and 22(OH)L3 are 20–30 higher than those reported for 20(OH)D3, consistent with the more efficient metabolism of lumisterol than D3 by human CYP11A1^13, 40^.

### Biological activity of lumisterol hydroxymetabolites in skin cells

**Figure 4:**
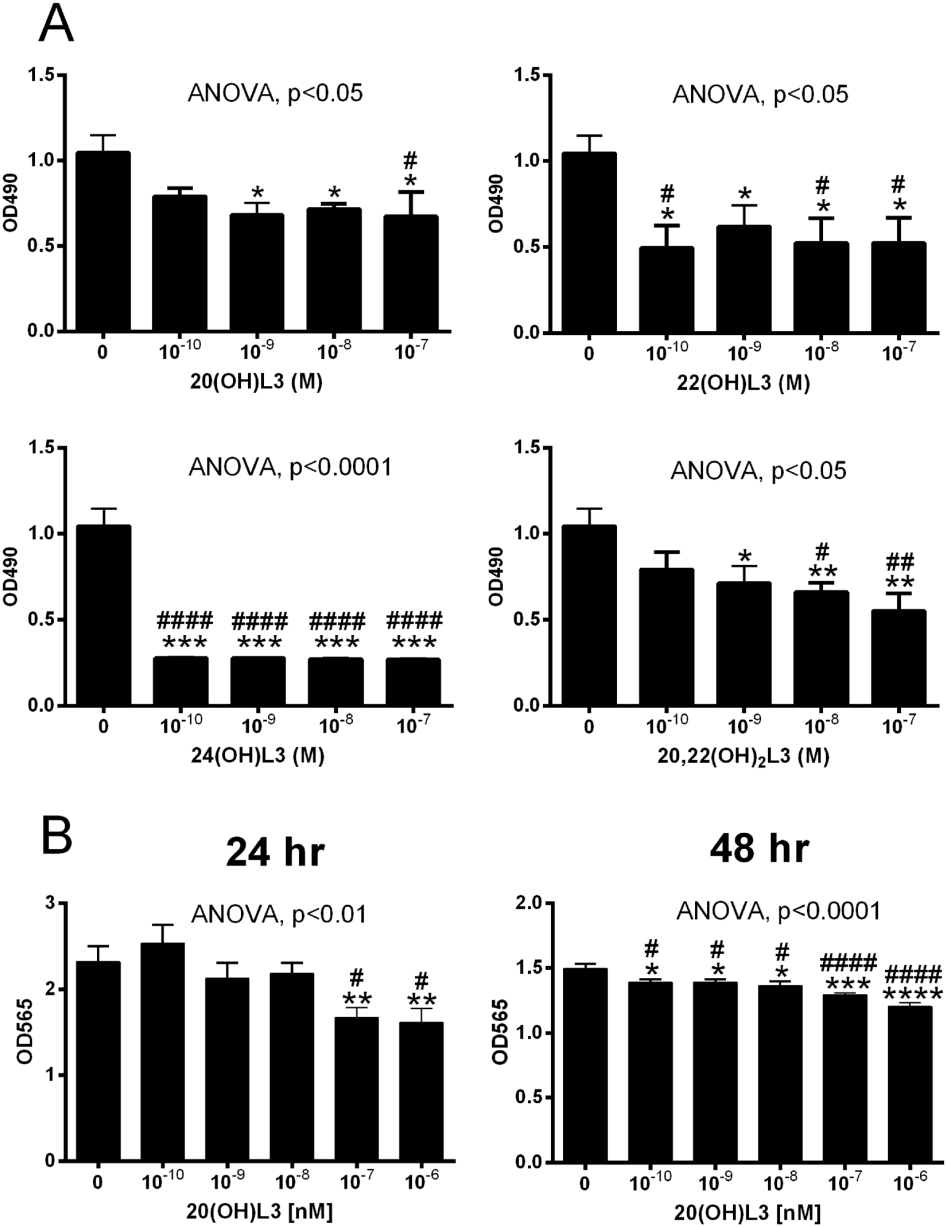
Inhibition of keratinocytes proliferation by 20(OH)L3, 22(OH)L3, 24(OH)L3 and 20,22(OH)_2_L3. (**A)**, MTS assay with HaCaT keratinocytes. The cells were synchronized by precincubation with serum-free media for 24 h, which was then replaced with DMEM plus 5% charcoal-treated FBS, and graded concentrations of hydroxylumisterols. After 48 h, the plates were used for the MTS assay performed at 490 nm. (**B)**, SRB assay with primary normal human epidermal keratinocytes. After 24 h of culture, fresh keratinocyte media containing graded concentrations of 20(OH)L3 were added. After 24 or 48 h, the plates were processed for SRB assays performed at 570 nm. Data represent means ± SE (n ≥ 3) where *p < 0.05, **p < 0.01, ***p < 0.001, and ****p < 0.0001 at student *t*-test; and #p < 0.05, ##p < 0.01 and ####p < 0.0001 at one-way ANOVA test and general ANOVA tests are shown.

20(OH)L3, 22(OH)L3, 24(OH)L3, and 20,22(OH)_2_L3 inhibited proliferation of epidermal immortalized (HaCaT) keratinocytes in a dose-dependent manner (Fig. 4A), with a potency similar to 1,25(OH)_2_D3 (not shown). 20(OH)L3 inhibited proliferation of human primary keratinocytes (Fig. 4B). These effects were similar to those described for 20(OH)D3 and 20,23(OH)_2_D3^22^.

The expression of a panel of genes by cultured human epidermal keratinocytes exposed to 20(OH)L3 was examined at the mRNA level (Table 2). Significantly, 20(OH)L3 stimulated the expression of genes encoding differentiation program markers (*INL, LOR, FLG, TGM1, KRT1, KRT5, KRT10*, and *KRT14*) and antioxidative enzymes (*CAT, GPX1, GSR, GSTP1, SOD1, SOD2, GCS, TXNRD1*, and *TRN*) (Table 2). Potential pro-differentiation and anti-oxidative effects were further confirmed by stimulation of INL and SOD2 (Mn-SOD) protein expression (supplemental Figure 5). Of additional interest is the increased expression of *BNIP3* (which is implicated in differentiation and maintenance of epidermal keratinocytes^46^), increased expression of *ICAM* (which plays a role in immune surveillance in basal cell carcinoma^47, 48^ and in wound healing^49^), and increased expression of gelsolin (which is implicated in apoptosis^50^, cancer, inflammation, infection and aging^51^). 20(OH)L3 also enhanced the expression of *CRH* and *URN*, which control skin responses to stress^52^, and enhanced the expression of *CYP1B1* which is involved in detoxification. Upregulation of the expression of the genes listed above indicates a role for hydroxylumisterols in the protective functions of the epidermis. With respect to immunomodulation and growth factors, regulation of these functions can be complex (Table 2). 20(OH)L3, while inhibiting the expression of *IL17A, INFA, INFB, INFG*, *TNFA, RANTES, IL6, RORA* and *RORC*) also inhibited *IL10* expression, while stimulating the expression of *IL1A, IL1B, CXCL8* and *IL22*. It stimulated the expression of *PDGFA*, *TGFB2* and *EGFR*, but inhibited that of *TGFA* and *TGFB1*. Complex regulation of the steroid-related systems is suggested by upregulation of *DHCR7* and selective inhibition of the expression of several steroidogenic genes. Moreover, 20(OH)L3 had stimulatory effects on the expression of genes encoding certain glycolytic enzymes (*ALDOA, LDHA*) while inhibiting *SLC2A1 (GLUT1)*. These data support the concept that CYP11A1-derived hydroxylumisterols can play diverse roles in the regulation of epidermal functions.

**Table 2:** Changes in gene expression in normal human epidermal keratinocytes treated with 0.01 µM 20(OH)L3 for 24 h.

Groups	Genes	Gene expression (fold change)		T-test p-value
		control	20(OH)L3	
Keratinocyte differentiation	INL	1 ± 0.37	28.38 ± 0.16	****
	LOR	1 ± 0.20	3.29 ± 0.26	***
	FLG	1 ± 0.18	2.01 ± 0.23	**
	TGM1	1 ± 0.17	2.54 ± 0.22	****
	KRT1	1 ± 0.17	7.18 ± 0.22	****
	KRT5	1 ± 0.26	2.58 ± 0.22	****
	KRT10	1 ± 0.18	4.91 ± 0.27	****
	KRT14	1 ± 0.34	4.39 ± 0.23	****
	ICAM	1 ± 0.39	18.25 ± 0.24	****
Antioxidative functions	CAT	1 ± 0.33	2.01 ± 0.14	**
	GPX1	1 ± 0.29	2.22 ± 0.17	**
	GSR	1 ± 0.35	44.32 ± 0.28	****
	GSTP1	1 ± 0.31	3.59 ± 0.12	****
	SOD2^#^	1 ± 0.30	1.47 ± 0.06	*
	SOD1	1 ± 0.30	1.46 ± 0.13	*
	GCLC	1 ± 0.30	3.62 ± 0.25	***
	TXNRD1	1 ± 0.35	8.21 ± 0.15	****
	TXN	1 ± 0.30	12.47 ± 0.20	****
Detoxification	CYP1B1	1 ± 0.29	2.19 ± 0.31	**
Gelsolin	GSN	1 ± 0.46	12.24 ± 0.15	****
CRF-related peptides	CRH	1 ± 0.36	7.62 ± 0.26	****
	UCN	1 ± 0.29	2.91 ± 0.41	*
Immune functions	IL1A	1 ± 0.32	2.58 ± 0.13	***
	IL1B	1 ± 0.32	1.50 ± 0.14	*
	IL2	1 ± 0.41	0.54 ± 0.30	NS
	IL4	1 ± 0.37	0.14 ± 0.19	*
	IL5	1 ± 0.41	0.81 ± 0.29	NS
	IL6	1 ± 0.34	0.27 ± 0.16	*
	CXCL8	1 ± 0.30	2.09 ± 0.13	**
	IL10	1 ± 0.31	0.08 ± 0.19	**
	IL17A	1 ± 0.37	0.09 ± 0.22	*
	IL22	1 ± 0.27	1.64 ± 0.23	*
	CCL5	1 ± 0.39	0.38 ± 0.25	*
	INFΝΑ1	1 ± 0.32	0.12 ± 0.25	**
	INFNΒ1	1 ± 0.29	0.51 ± 0.14	*
	INFNG	1 ± 0.40	0.19 ± 0.15	*
	TNFA	1 ± 0.39	0.10 ± 0.20	*
	CD14	1 ± 0.35	0.15 ± 0.21	*
	BCL2	1 ± 0.29	0.73 ± 0.13	NS
	BNIP3	1 ± 0.31	24.82 ± 0.17	****
	RORΑ	1 ± 0.29	0.16 ± 0.15	**
	RORC	1 ± 0.3	0.15 ± 0.32	*
Growth factors	PDGFA	1 ± 0.39	3.17 ± 0.29	***
	TGFA	1 ± 0.29	0.10 ± 0.12	**
	TGFB1	1 ± 0.30	0.33 ± 0.25	*
	TGFB2	1 ± 0.33	1.79 ± 0.16	*
	EGFR	1 ± 0.36	1.98 ± 0.28	*
	VEGFA	1 ± 0.33	1.14 ± 0.14	NS
Steroids related	DHCR7	1 ± 0.31	2.84 ± 0.23	***
	NR3C1	1 ± 0.42	0.53 ± 0.21	NS
	CYP17A1	1 ± 0.5	0.16 ± 0.27	*
	CYP21 A2	1 ± 0.34	0.13 ± 0.33	*
	HSD3B1	1 ± 0.29	0.05 ± 0.17	*
	HSD11B1	1 ± 0.29	1.4 ± 0.27	NS
	HSD11B2	1 ± 0.52	1.17 ± 0.28	NS
	CYP11A1	1 ± 0.38	0.33 ± 0.19	NS
	CYP11B1	1 ± 0.35	0.75 ± 0.26	*
	POMC	1 ± 0.30	0.22 ± 0.19	**
Cellular metabolism	ALDOA	1 ± 0.29	4.37 ± 0.14	****
	HIF1A	1 ± 0.39	1.32 ± 0.36	NS
	SLC2A1	1 ± 0.29	0.33 ± 0.17	*
	HK2	1 ± 0.29	1.13 ± 0.20	NS
	LDHA	1 ± 0.32	6.57 ± 0.12	****
	NHEI	1 ± 0.34	3.95 ± 0.16	***

qPCR data were generated from input cDNA using a Cyber Green Master Mix, performed in 384-well plates on a Applied Biosystems instrument at 95 °C for 5 min and then 40 cycles of 95 °C for 15 s and 55 °C for 30 s^45^. Relative gene expression was calculated using a ΔΔ*C*_*t*_ method using Cyclophylin B as internal control. Sequences of primers are in supplemental Table 2. Cyclophilin B was used as internal control. Data represent mean ± SD; n = 3. Significance was analyzed using student-*t* test, *P < 0.05; **P < 0.01; ***p < 0.001. ^#^Values are presented for 10^−7^ M 20(OH)L3. ALDOA: Aldolase A, BCL2: B-cell lymphoma 2, BNIP3: BCL2 adenovirus E1B interacting protein, CAT: catalase, CCL5: C-C motif chemokine ligand 5, CD14: cluster of differentiation 14, CRH: corticotropin releasing hormone, CXCL8: C-X-C motif chemokine ligand 8, CYP21 A2: cytochrome P450 family 21 subfamily A member 2, DHCR7: sterol ∆7-reductase, EGFR: epidermal growth factor receptor, FLG; fillagrin, GCLC: glutamate-cysteine ligase catalytic subunit,, GPX1: glutathione peroxidase 1, GSN: gelsolin, GSR: glutathione-disulfide reductase, GSTP1: glutathione S-transferase pi 1, HIF1A: hypoxia inducible factor 1 alpha subunit, HK2: hexokinase, HSD3B1: hydroxy-delta-5-steroid dehydrogenase, 3 beta- and steroid delta-isomerase 1, ICAM: Intracellular adhesion molecule, IL: interleukin, IFNA1: interferon alpha 1, INFNB1: interferon beta 1, IFNG: interferon gamma, INL: involucrin, KRT: cytokeratin,, LDHA: lactate dehydrogenase A, LOR: loricrin, NHEI: Na/H exchange inhibitor, NR3C1: nuclear receptor subfamily 3 group C member 1, PDGFA: platelet derived growth factor-α, POMC: proopiomelanocortin, RORA: RAR related orphan receptor A, RORC: RAR related orphan receptor C, SLC2A1: solute carrier family 2 member 1 also known as GLUT1, SOD: superoxide dismutase (SOD1: Cu/Zn-SOD; SOD2: Mn-SOD), TGFA: transforming growth factor alpha, TGM1: transglutaminase, TNF: tumor necrosis factor alpha, TXNRD1: thioredoxin reductase 1, TXN: thioredoxin, UCN: urocortin, VEGFA: vascular endothelial growth factor A.

**Figure 5:**
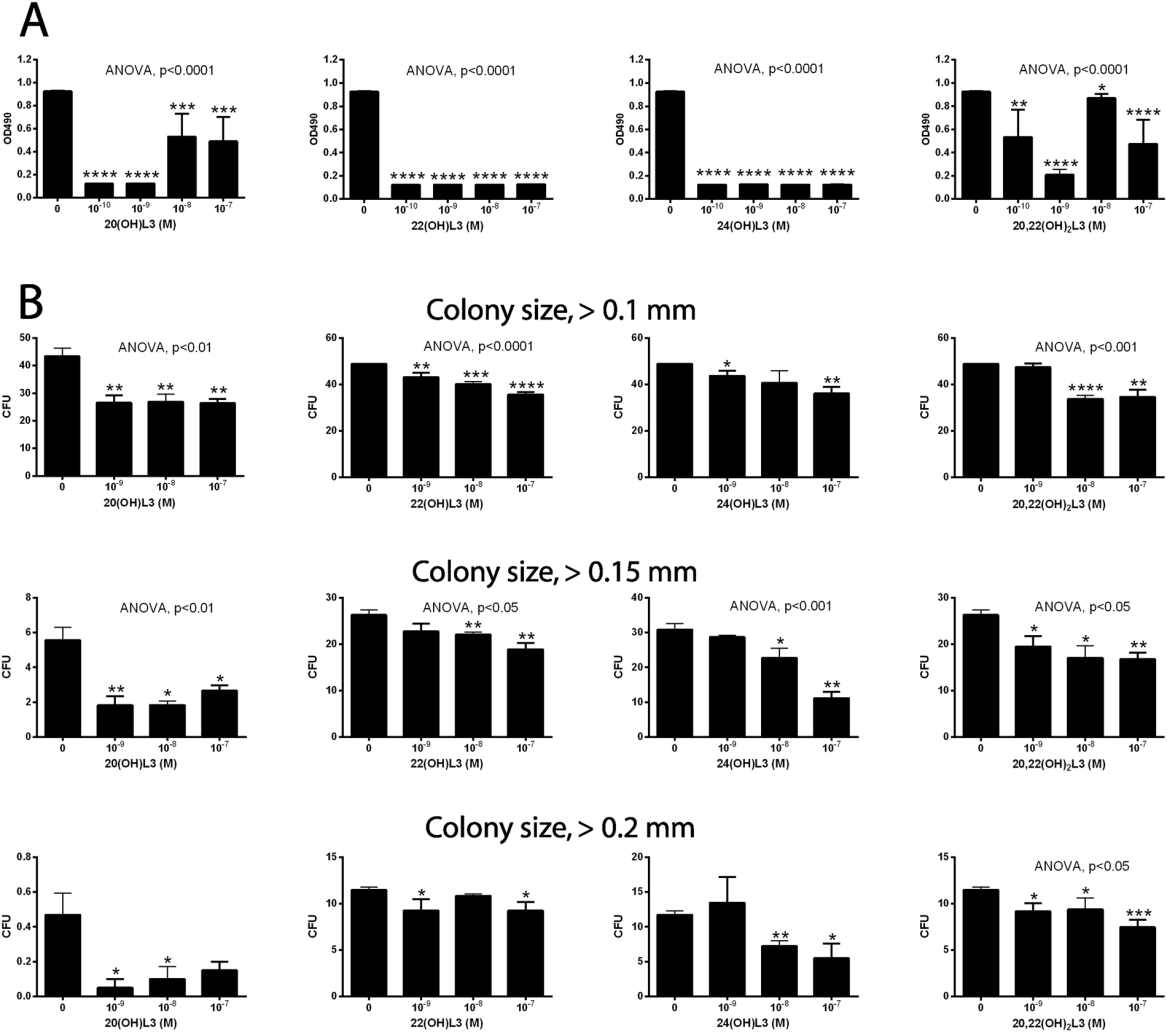
Inhibition of SKMEL-188 human melanoma growth by 20(OH)L3, 22(OH)L3, 24(OH)L3 and 20,22(OH)_2_L3. (**A**), Inhibition of proliferation in monolayer assed by MTS assay. After 24 h of culture, the cells were exposed to graded concentrations of hydroxylumisterols suspended in Ham’s F10 plus 10% charcoal-treated FBS. After 48 h, the plates were used for MTS assay performed at 490 nm. (**B**), Inhibition of growth in soft agar (anchorage independent growth). Melanoma cells were suspended in medium containing 0.4% agarose (American Bioanalytical, Natick, MA) and 5% charcoal-treated FBS, and seeded at 1,000 cells/well in a 0.8% agar layer in 24-well plates and treated with the graded concentrations of the listed compounds which were freshly added every 72 h over 13 days^54^. The colonies stained with MTT reagent (Promega, Madison, WI) were analyzed using the Cytation 5 Cell Imaging Multi-Mode Reader in three different z-planes and scored using Gen5 software^54^. Data represent means ± SE (n ≥ 3) where *p < 0.05, **p < 0.01 and ***p < 0.001 by the student *t*-test, and general ANOVA tests are shown.

Since melanoma still represents a clinically challenging problem^53^, we evaluated the anti-melanoma activity of the hydroxylumisterols (Fig. 5A,B). 20(OH)L3, 22(OH)L3, 24(OH)L3, and 20,22(OH)_2_L3 markedly inhibited cell proliferation (Fig. 5A) with the structurally related 20(OH)Chol having no significant effect (not shown). At the same time point (48 h of incubation) hydroxylumisterols had no effect on the proliferation of normal melanocytes, with moderate inhibitory effects seen at 72 h, but only for 20(OH)L3 and 22(OH)L3 (supplemental Figure 6). All hydroxylumisterols inhibited the anchorage-independent melanoma growth in soft agar (Fig. 5B), indicating their antitumorogenic potential. They did not affect melanin production by melanoma cells (not shown). The effects on proliferation are similar to those described for CYP11A1-derived hydroxyvitamin D3-derivatives, which also showed strong anti-melanoma effects with weak or absent effects on normal melanocytes^55, 56^. In addition, our previous studies demonstrated *in vitro* anti-melanoma activity of mono and dihydroxy pL compounds^33–36^. Thus, the novel hydroxyderivatives of lumisterol are good candidates for further testing of their therapeutic utilities using animal models of melanoma and patient-derived orthotopic xenografts (PDOX) models^57–59^.

In normal human dermal fibroblasts we found moderate inhibition of cell proliferation by 20(OH)L3 and 22(OH)L3, and to a lesser degree by 24(OH)L3, but not for 20,22(OH)_2_L3 (supplemental Figure 7). 20(OH)L3, 22(OH)L3 and 20,22(OH)_2_L3 also inhibited basal and TNFα-induced NFκΒ transcriptional activity in 3T3 fibroblasts (supplemental Figure 8). These studies identify fibroblasts as an additional target for regulation by the hydroxylumisterols, which supplements our previous finding that 17,20(OH)_2_pL shows antifibrogenic activity^39^.

### Hydroxylumisterols can interact with RORα and RORγ

#### RORγ and RORα-mediated transactivation assays

RORα and RORγ are expressed in the human skin^24, 25^, while L3 analogs are structurally very similar to sterols that are examples of native ligands for RORs^26, 60, 61^. Therefore, we tested their effects on RORs using cell based and *in vitro* assays. First, using a previously described Tet-on CHO cell reporter system for analysis of RORγ and RORα-mediated transactivation^24^, we compared the inverse agonist activity of 20(OH)L3 with that of its structurally related D3 and sterol derivatives. Supplemental Figure 9A shows that 20(OH)L3 was the most potent inhibitor of RORγ-induced transcriptional activity, being less active on RORα. Additional tests on skin derived cells transfected with the RORE-LUC reporter showed a dose-dependent inhibition of luciferase activity by 20(OH)L3 (Supplemental Figure 9B). Interestingly, 20(OH)7DHC, a potential precursor to 20(OH)L3, was less potent (Supplemental Figure 9b).

**Figure 6:**
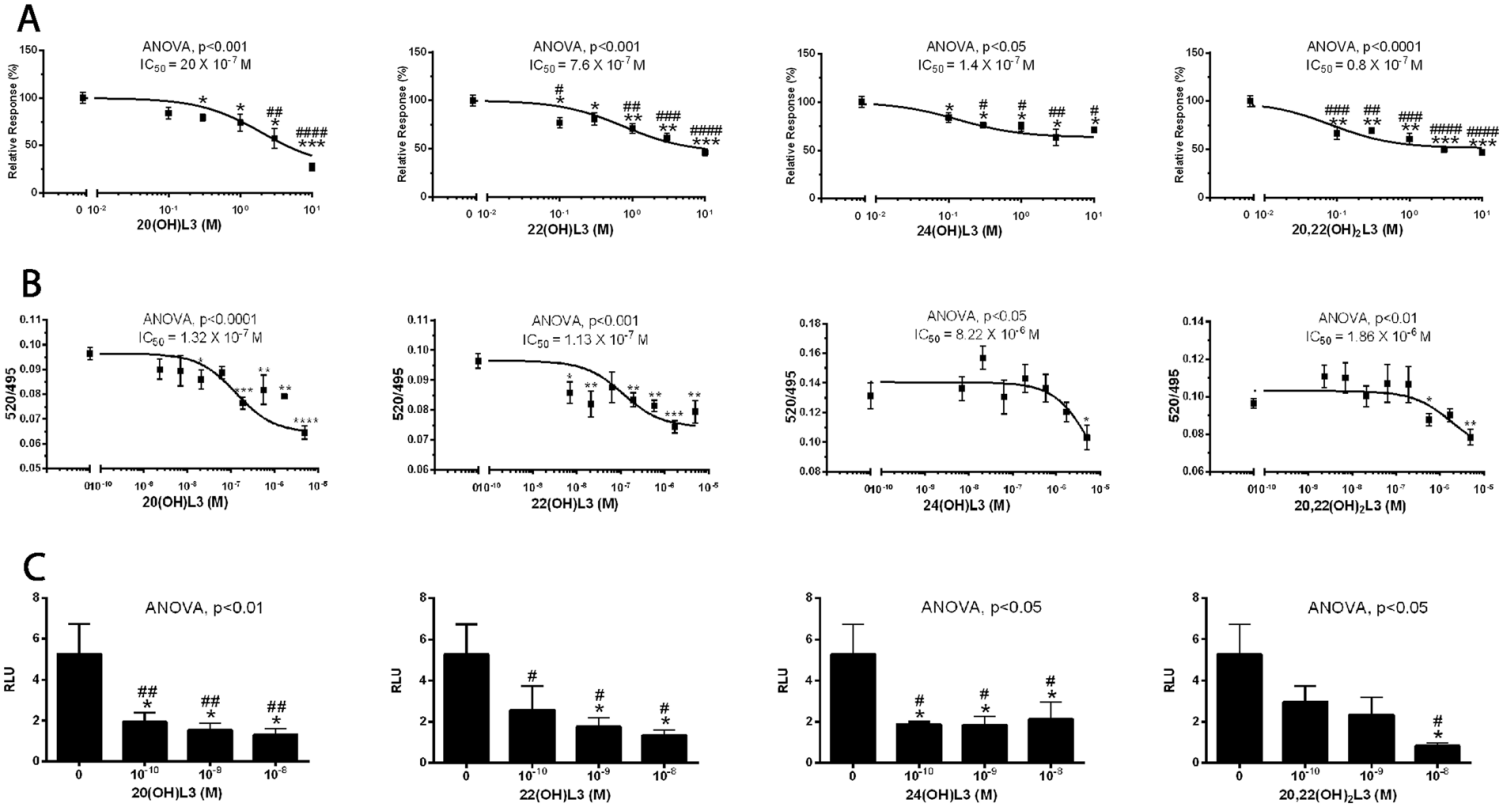
Modulation of RORα and RORγ activities by 20(OH)L3, 22(OH)L3, 24(OH)L3 and 20,22(OH)_2_L3. (**A**), RORγ transactivation assay in Tet-on CHO cells. To induce expression of RORγ protein expression, CHO cells were treated with 1 μM doxycycline for 24 h. To measure the transactivation the cells were treated with graded concentrations of the hydroxylumisterols listed, and the RORE-mediated activation of the luciferase reporter activity was assayed with a Luciferase Assay Substrate kit (Promega) as described previously^24^. Assays were performed in triplicate. (**B**), RORα coactivator assay using LanthaScreen TR-FRET RORα Coactivator kit assay. RORα-LBD was added to graded concentrations of hydroxylumisterols followed by the addition of a mixture of peptide (TRAP220/DRIP2) and antibody (Tb-anti-GST). The reaction mixture was incubated at room temperature for 2 h and the TR-FRET ratio was calculated by dividing the fluorescein emission at 520 nm by the Terbium emission at 495 nm using Synergy neo2 (BioTek Instruments, Inc., Winooski, VT). Data represent means ± SE (n ≥ 3) where ^*^p < 0.05, ^**^p < 0.01 and ^***^p < 0.001 student t-test; ^#^p < 0.05, ^##^p < 0.01, ^###^p < 0.001 and ^####^p < 0.0001 by one-way ANOVA and general ANOVA tests are shown. (**C**), RORE luciferase assay in HaCaT keratinocytes. The cells were cotransfected with the reporter plasmids pGL4.27-(RORE)_5_ and phRL-TK (Promega) using Lipofectamine (Invitrogen, Carlsbad, CA) following the manufacturer’s protocol. After transfection, the cells were treated with hydroxylumisterols for 48 h. Luciferase reporter activity was measured using the Dual-Luciferase Reporter Assay System (Promega, Madison, WI). Firefly and Renilla signals were read using Cytation 5 (BioTek Instruments, Inc., Winooski, VT), and the ratios were calculated.

Next, we tested the effects of 20(OH)L3, 22(OH)L3, 24(OH)L3, and 20,22(OH)_2_L3 on RORγ transcriptional activity using the Tet-on CHO cell system (containing the Luc reporter, pGL4–27–5xRORE)^24^, and interaction with RORα using the LanthaScreen TR-FRET RORα Coactivator assay (Fig. 6A,B). We found that the hydroxylumisterols inhibited doxycycline induced RORγ transcriptional activity in a dose-dependent manner in the Tet-on CHO system (Fig. 6A). Furthermore, 20(OH)L3, 22(OH)L3, 24(OH)L3, and 20,22(OH)_2_L3 decreased the affinity of the co-activator peptide for RORα–LBD (ligand binding domain) (Fig. 6B). Finally, using HaCaT keratinocytes transfected with the reporter plasmid pGL4.27-(RORE)_5_, we observed a significant inhibition of RORE-LUC reporter activity by 20(OH)L3, 22(OH)L3, 24(OH)L3, and 20,22(OH)_2_L3 (Fig. 6C).

These functional and *in vitro* assays clearly establish interaction of 20(OH)L3, 22(OH)L3, 24(OH)L3 and 20,22(OH)_2_L3 with RORs in a manner expected for reverse agonists on these receptors^24, 26, 61^.

#### Docking results for RORα and γ

The hydroxylumisterols and related compounds listed in Table 3 were docked into crystal structures of RORα and RORγ using the Glide docking in extra-precision mode (XP) (Schrödinger package). Preparation of structures and the docking protocol are described in the Supplemental information. Docking results are presented below.

**Table 3:** Glide XP scores of compounds docked into RORα, γ and VDR crystal structures. VDR sites used: non-genomic pocket (VDR – A), genomic pocket (VDR – G). ND = Not Docked.

Compound	Structure	Glide XP Score	RORγ	VDR - A	VDR - G
		RORα			
20(OH)chol	A: R1 = OH	−11.60	−12.17	−10.01	−9.78
20(OH)7DHC	B: R1 = OH	−12.26	−12.65	−10.79	−9.57
7DHC	B: R1 = H	−12.11	−12.66	−10.80	−10.37
cholesterol	A: R1 = H	−11.92	−12.49	−10.31	−9.99
lumisterol	C: R1 = R2 = R3 = H	−11.40	−11.72	−11.99	−9.80
20-hydroxylumisterol	C: R1 = OH, R2 = R3 = H	−11.43	−11.36	−12.30	−9.62
22-hydroxylumisterol	C: R1 = R2 = H, R2 = OH	−10.91	−11.09	−12.93	−9.82
24-hydroxylumisterol	C: R1 = R2 = H, R3 = OH	−11.55	−11.99	−13.74	−9.63
20,22-dihydroxylumisterol	C: R1 = R2 = OH, R3 = H	−12.25	−11.96	−12.72	−9.12
pregnalumisterol	D: R1 = R2 = H	−9.69	−9.76	ND	ND
17-hydroxy-pregnalumisterol	D: R1 = OH, R2 = H	−10.30; −9.67	−10.61; −8.94	ND	ND
17,20-dihydroxy-pregnalumisterol	D: R1 = R2 = OH				
	20 *S*	−11.37	−11.57	ND	ND
	20 *R*	−11.13	−12.29		
1α,25-dihydroxylumisterol		ND	ND	−12.82	−11.40
1α,25-dihydroxyvitamin D3		−10.30	−10.30	−12.24	−15.07
melatonin		−6.69	−6.96	−6.91	−6.59

**Figure 7:**
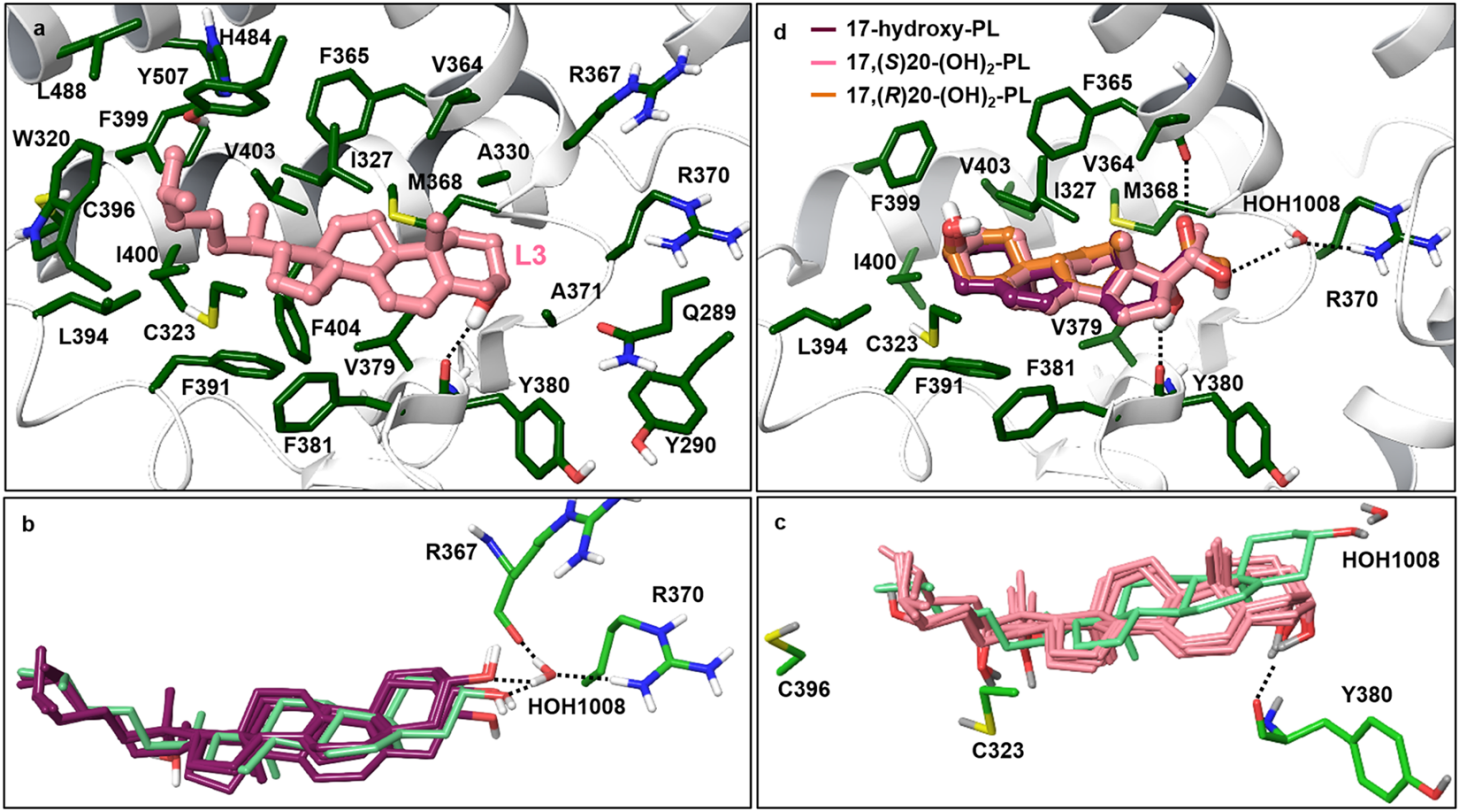
Docking results using the crystal structure of RORα. (**a**). The ligand binding site of RORα with the docked pose of lumisterol. Residues mapping the active site are shown; protein carbons are colored dark green, ligand carbons pink, all other atoms by atom type (O red, N blue, S yellow, H white). The dashed line indicates hydrogen bonding between lumisterol and Tyr380 (2.1 Å distance between interacting atoms). (**b-c**). The overlap of docked poses is illustrated in the RORα binding site in comparison with the co-crystallized 20-hydroxycholesterol. (**b)** Cholesterol analogs (purple carbons) and (**c**) Hydroxylumisterol analogs (pink carbons). The co-crystallized cholesterol is shown with light green carbons. Only residues that may contribute to polar interactions are shown; dashed lines indicate hydrogen bonds. (**d)** The ‘flipped’ pose of hydroxylated pregnalumisterol (pL) analogs binding to RORα. Carbon atoms of ligands are color-coded as shown; protein carbons are dark green (all other atoms are colored by atom type). Hydrogen bonding interactions are shown with dashed lines. Interactions contributed by the 20-OH group of 17, 20(OH)_2_pL enantiomers: (*S*)20-OH forms a water-bridged hydrogen bond with R370; (*R*)20-OH participates in hydrogen bonding with the backbone carbonyl of Val364.

*RORα*. The RORα ligand binding site with docked L3 is illustrated in Fig. 7a. The binding site of RORα is largely hydrophobic and shows structural complementarity with L3. Desolvation and formation of favorable non-polar interactions is the most significant contributor to the binding of L3. Similarly to L3, docked poses of (OH)_n_L3 compounds listed in Table 3 form non-polar contacts that are analogous to those present in the crystal structure between cholesterol and RORα residues. As illustrated in Fig. 7b,c, docked poses of cholesterol analogs and the (OH)_n_L3 series (Table 3) are approximately overlapping with the cholesterol co-crystallized in the RORα binding site. The residues displayed are predicted to contribute polar contacts with the ligands. L3 forms a hydrogen bonding interaction between its 3-hydroxyl group and the carbonyl backbone of Tyr380. Polar interactions of cholesterol analogs involve hydrogen bonding through a tightly bound crystal water, as shown in Fig. 7b. In the (OH)_n_L3 series, all analogs hydrogen bond with the backbone carbonyl of Tyr380, an interaction shared among lumisterol analogs. Docked poses suggest that Cys323 and Cys396 may contribute to polar interactions as hydrogen bond donors to 22-hydroxyl and 24-hydroxyl groups, respectively. PL has a markedly lower docking score for RORα due to lack of a side chain, which in other lumisterol analogs contributes to binding through non-polar interactions. For the hydroxylated pL analogs, 17(OH)pL and 17,20(OH)_2_pL, two possible poses were obtained: one that is similar to the (OH)_n_L3 series and a second pose adopting the opposite or ‘flipped’ orientation, as illustrated in Fig. 7d. Compared to L3, these compounds are shorter, more polar and are capable of forming hydrogen-bonding interactions in either orientation. In the case of 17,20(OH)_2_pL, the ‘flipped’ pose may be more likely since it predicts an additional hydrogen bonding interaction via the 20-hydroxyl group (Fig. 7d). Interestingly, the theoretically deduced flipped positions for hydroxyl-pL compounds are further substantiated by their apparent agonistic activity on RORα as shown in supplemental Figure 10. The more favorable hydrogen bonding contribution as reflected in the improved Glide XP scores of 17,20(OH)_2_pL compared to pL (Table 3) is also supported by its higher potency in comparison to pL (supplemental Figure 10). Overall, docking results predict favorable binding of the (OH)_n_L3 series and hydroxylated pL analogs to RORα.

**Figure 8:**
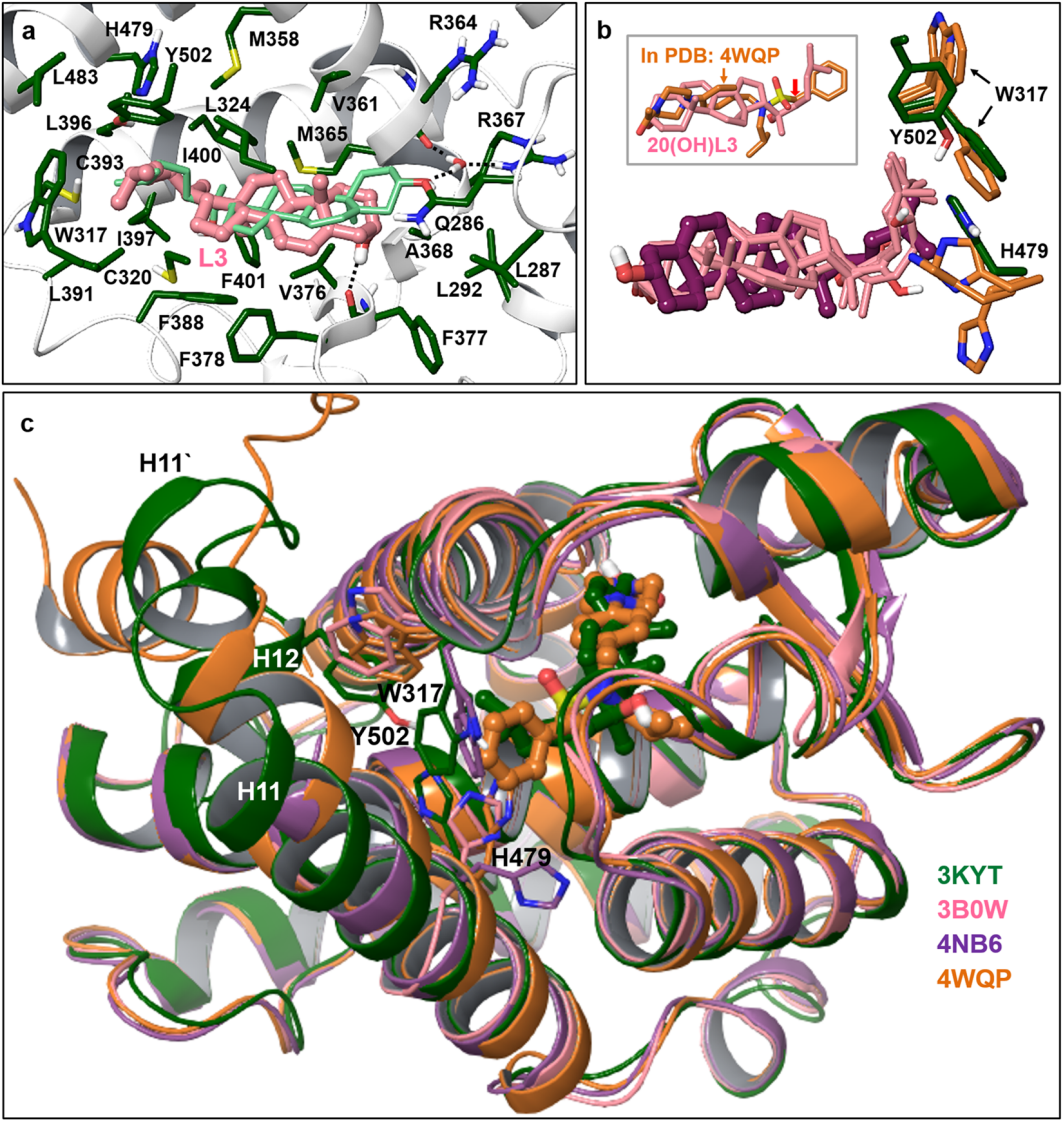
Docked poses in the RORγ binding site and comparison with inverse agonist bound crystal structures. (**a**) Docked lumisterol in the RORγ active site is shown in comparison with the co-crystallized 20-hydroxycholesterol (light green colored carbons). Similar view angle and the same color coding is used as in case of RORα (Fig. 7a). Hydrogen bonding interactions are indicated with dashed lines. (**b**) The overlap of docked poses is illustrated for hydroxylumisterols (20(OH)L3, 22(OH)L3, 24(OH)L3, 20,22(OH)_2_L3) with pink color carbons while cholesterol is shown with thick bonds and carbons colored maroon. Key residues involved in inverse agonism of co-crystallized ligands are shown only. Residues in the RORγ crystal structure used in our docking study are displayed with dark green carbons (PDB code 3KYT); residues in RORγ structures co-crystallized with inverse agonist ligands are shown with brown colored carbons (PDB codes 3B0W, 4NB6, 4WQP). Using the same view angle and orientation the inset illustrates 20-hydroxylumisterol in comparison with an inverse agonist ligand co-crystallized in RORγ (PDB code 4WQP), shown with brown colored carbons. The analog of this ligand lacking the one-carbon linker marked with a red arrow is a RORγ agonist. (**c**) RORγ crystal structures with inverse agonists are aligned onto the structure with PDB code 3KYT. The three key residues (as in Fig. 8b) are also shown, along with co-crystallized ligands from two structures: PDB codes 3KYT and 4WQP. Font colors of PDB codes listed correspond to the coloring of secondary structures and carbon atoms of co-crystallized ligands shown.

*RORγ*. Analogously to docking results obtained at RORα, docked poses of cholesterol analogs in RORγ are overlapping closely with the co-crystallized 20-hydroxycholesterol. Similarly to the co-crystallized ligand, the 3-hydroxyl group in all docked cholesterol analogs participates in hydrogen bonding with the side chain of Gln286 and forms a water-bridged hydrogen bond with Arg367. As in RORα, the active site of RORγ is predominantly hydrophobic and ligand binding is primarily driven by non-polar interactions and desolvation of non-polar groups. Docking of the (OH)_n_L3 series (Table 3) in the RORγ binding site predicts analogous non-polar contacts to those formed by the co-crystallized 20(OH)Chol ligand, as illustrated for lumisterol as an example in Fig. 8a. The hydrogen bonding interaction shown between L3 and the backbone carbonyl of Phe377 is shared among all docked analogs in the (OH)_n_L3 series. Docking of pregnalumisterols into RORγ suggested less favorable binding (less favorable docking scores) compared to the lumisterol series due to the missing non-polar interactions in the side chain region. Two possible and opposite orientations have been predicted for 17(OH)pL. Most favorably scoring poses of 17,20(OH)_2_pL enantiomers are flipped by approximately 180° compared to lumisterol poses, which allows hydrogen-bonding interactions with the carbonyl backbone of Phe377 and the His479 side chain. The more favorable docking score of 17,20(OH)_2_pL compared to pL is due to the formation of additional hydrogen bonding interactions. Thus, favorable binding of the (OH)_n_L3 series and hydroxylated pL is also predicted for RORγ, similarly to docking results obtained for the same series for RORα.

Co-crystallized inverse agonists in RORγ X-ray structures show disruption of a key hydrogen bonding interaction between H479 and Y502 while this interaction in agonist co-crystal structures is undisturbed or further stabilized by agonist ligands. W317 has also been identified as a key residue accessible to ligands that may contribute to propagating the effects of inverse agonists. Conformational changes triggered by the binding of inverse agonists are destabilization of helices 11, 11, 12, which leads to lack of coactivator recruitment. We have not considered using these inverse agonist bound RORγ structures for lumisterol series docking since these ligands are significantly different in terms of shape and polarity from the lumisterol scaffold and ligand induced effects are important to reproduce optimal non-polar contacts in the RORγ binding site. Figure 8b illustrates the conformation of key residues for functional activity from three RORγ crystal structures with distinct inverse agonists bound (PDB codes 3B0W, 4NB6, 4WQP), based on their alignment onto the structure with PDB code 3KYT. Docked poses of the (OH)_n_L3 series are also displayed in comparison with the docked cholesterol (which is shown with thick bonds). In the docked pose of 24(OH)L3 the 24-hydroxyl group is close enough for hydrogen bonding interaction with H479. However, as shown in Fig. 8b, distinct conformations of the H479 side chain are possible in close proximity to both 22- and 24-hydroxyl groups of the (OH)_n_L3 series (while Y502 in helix 12 is disordered). Further, the hydroxylated aliphatic chain in the lumisterol series is overlapping with a functionally important region of the inverse agonist shown in the inset of Fig. 8b. This ligand induces flipping of the W317 side chain, which leads to displacement of Y502. Removal of a one-carbon linker group (marked with arrow) converts this inverse agonist into an RORγ agonist that shows no steric clashes with W317 (PDB: 4WPF). Alignment of the RORγ structures with inverse agonists onto RORγ with co-crystallized 20(OH)Chol (PDB: 3KYT) shows close overlap except for helices 11, 11’ and 12 (Fig. 8c). Residues affected by these conformational differences that are accessible to docked lumisterol analogs are in the region of the key residues W317, H479, H502.

Considering the close proximity of hydroxyl group substituents in the (OH)_n_L3 series to the functionally important H479 we hypothesize that a possible mechanism of inverse agonism of these ligands is through hydrogen bonding with H479 and disruption of the H479 – Y502 interaction.

#### Summary remarks on RORs

Thus, L3 and its hydroxyderivatives show favorable docking scores and binding poses in the LBD of RORγ and RORα, similar to natural ligands (sterols and hydroxysterols). The *in silico* predictions provide additional support for the functional studies on RORs and, as reported in Fig. 6 and supplemental Figure 10, that 20(OH)L3, 22(OH)L3, 24(OH)L3, and 20, 22(OH)_2_L3 can interact and modify the activities of RORα and RORγ.

### Vitamin D receptor

Functional testing of binding to the VDR has shown that the hydroxylumisterols lack any effect on VDRE- transcriptional activity in HaCaT cells (Supplemental Figure 11) and do not bind to the genomic LBD of the VDR using the LanthaScreen TR-FRET competition kit (not shown). Docking of hydroxylumisterols into the genomic (G)-pocket of the VDR gave significantly poorer scores than those for 1,25(OH)_2_D3 and 1,25(OH)_2_L3 (Table 3). Therefore, we conclude that CYP11A1-derived hydroxylumisterols are not involved in the regulation of genomic VDR activity.

Surprisingly, we have found that the hydroxylumisterols stimulated translocation of the VDR from the cytoplasm to the nucleus but required relatively high concentrations (Supplemental Figure 12). However, these effects were significantly lower in comparison to 1,25(OH)_2_D3 and other hydroxyderivatives of vitamin D^23, 62^. Therefore, we performed molecular modeling to predict whether the hydroxylumisterols can bind to the non-genomic site (A-pocket) of the VDR, a binding site for 1,25(OH)_2_L3^63, 64^, using docking simulations (see description in the Supplemental information). The resulting docked poses show favorable interactions in the A-pocket with Glide XP scores being comparable to or better than those for 1,25(OH)_2_L3 and 1,25(OH)_2_D3 (Supplemental Figures 13 and 14) (Table 3). Therefore, it is possible that the CYP11A1-derived hydroxylumisterols might act on the non-genomic A-pocket of the VDR. This exciting possibility deserves future investigation.

## Conclusions

Lumisterol was previously considered to be a metabolically inactive end product of 7DHC exposure to high UVB energy, providing an explanation of why UVB-induced production of pre-D3 does not lead to systemic D3 intoxication^5, 7^. The current study shows that this traditional view must be revised, since lumisterol not only enters the systemic circulation (having serum levels at ~5 × 10^−8^ M) but it can be hydroxylated *in vivo* by CYP11A1. The approximate serum concentrations of 20(OH)L3(~2.5 × 10^−8^ M) and of 22(OH)L3 (~0.8 × 10^−8^ M) are lower than that of 25(OH)D3, while that of 20,22(OH)_2_L3 (~0.3 × 10^−8^ M) is higher than that for 1,25(OH)_2_D3 (~10^−10^ M).

The CYP11A1-derived hydroxylumisterols inhibit skin cell proliferation in a cell-type dependent fashion with pronounced effects on keratinocytes, and show anti-melanoma activity as well. 20(OH)L3, as a representative hydroxylumisterol, also stimulates expression of genes associated with keratinocyte differentiation and anti-oxidative programs. The functional data presented on the hydroxylumisterols, in conjunction with the previously described biological effects of pL analogs^34, 36, 39, 65^, suggest that the novel lumisterogenic pathway might be involved in the regulation of cutaneous homeostasis. Their discovery also opens up exciting new areas for future research such as studies on the role of lumisterol derivatives in barrier function, photoprotection, skin cancer and studies on their possible therapeutic or adjuvant utility in the management of melanoma.

The cell based and *in vitro* analyses of activities of RORs, supported by molecular modeling, demonstrate that the hydroxylumisterols can act as ligands on RORα and RORγ. However, it is unlikely that they interact with the genomic site of the VDR.

In summary, this study reveals that a CYP11A1–mediated pathway of lumisterol metabolism occurs *in vivo*, the products of which have phenotypic/biological activities determined by their structure and cellular target.

## Materials and Methods

### Source of lumisterol derivatives

Lumisterol, 7DHC, D3 and 1,25(OH)_2_D3 were obtained from Sigma-Aldrich (St. Louis, MO). 20(OH)7DHC, 20(OH)L3, pL, 17(OH)pL and 17,20(OH)_2_pL were synthesized as previously described^32, 34, 43^; while 22(OH)L3, 20,22(OH)_2_L3 and 24(OH)L3 were produced from L3 enzymatically using purified bovine CYP11A1^40^. These compounds were purified by reverse-phase HPLC; their structures and purities were determined by NMR and mass spectrometry^32, 34, 40, 43^.

### Use of tissues and serum samples

Collection of human or pig tissue and serum samples was approved by the Institutional Review Board (IRB) (Human Subject Assurance Number 00002301) and the Institutional Animal Care and Use Committee (IACUC) (Animal Welfare Assurance Number A3325–01) at the University of Tennessee Health Science Center (UTHSC) with details of protocols and collection of the samples previously described^19^. Pig adrenals were obtained from a female Landrace cross Large White pig, 2 years old. All methods were performed in accordance with the relevant guidelines and regulations, see below. Tissues or sera were extracted with organic solvents and stored at −80 °C^19^, prior to aliqots being taken for LC/MS analyses. These same samples have been used previously for vitamin D metabolism studies, as detailed in ref. 19.

Human skin samples (n = 13) were collected in Memphis during 2013 and 2014 from 7 males and 6 females that comprised 6 African-Americans (AA) and 7 Caucasians (C) whose age ranged from 30 to 90 years^66^. Human sera were collected on March 28, 2014 in Memphis from 13 volunteers (3 males and 10 females) comprising 12 C and 1 Hispanic who were 25–61 years old. The use of human skin was approved by the IRB at the UTHSC as an exempt protocol #4 (Dr. A. Slominski, P.I.). This protocol was classified for exempt status under 45CFR46.102 (f) in that it does not involve “human subjects” as defined therein^19^. Collection of human serum was approved by IRB protocol #7526 (Dr. A. Postlethwaite, P.I.). Informed consent was obtained from all subjects involved in this study and the samples were deidentified as previously described^19^.

Skin (foreskins) from AA that would normally be discarded were used to establish primary cultures of keratinocytes, melanocytes and dermal fibroblasts, and was approved by the IRB at the University of Alabama Birmingham. This protocol was identified as not subject to FDA regulation and not Human Subject Research (IRB protocol N150915001 – Endocrine Functions of the Skin – revised version).

### Detection of lumisterol derivatives

Liquid chromatography and mass spectrometry (LC-MS) analyses followed protocols described previously^19^. For identification of lumisterol derivatives in extracted samples, we first separated the expected CYP11A1-derived hydroxylumisterols by HPLC using a Waters C18 column (250 × 4.6 mm, 5 μm particle size). The mobil)e phase used was a gradient of acetonitrile in water (40–100%) at a flow rate of 0.5 ml/min for 15 min followed by isocratic 100% acetonitrile for 30 min at a flow rate of 0.5 ml/min and then a flow rate of 1.5 ml/min for 20 min. Fractions with RT corresponding to the chemically or enzymatically synthesized standards (see section: *Source of lumisterol derivatives*) were collected and then subjected to UPLC [(Waters ACQUITY I-Class UPLC (ultra-performance liquid chromatography) system (Waters, Milford, USA)] on an Agilent Zorbax Eclipse Plus C18 column (2.1 × 50 mm, 1.8 µm particle size), connected to a Xevo™ G2-S qTOF (quadrupole hybrid with orthogonal acceleration time-of-flight) tandem mass spectrometer (Waters, Milford, USA) as detailed previously^19^. The mobile phase for UPLC comprised a gradient of methanol in water containing 0.1% formic acid (20–60% for 3 min then 60–100% for 1 min), followed by isocratic 99.9% methanol plus 0.1% formic acid for 2.1 min, all at flow rate of 0.3 ml/min.

For quantification, the concentrations of L3 and related compounds were directly analyzed by LC-MS using two different conditions of LC, as described in the Figure legends. For L3, D3 and 7DHC, a Waters Atlantis dC18 column (100 × 4.6 mm, 5 μm particle size) was used with a gradient of methanol in water (85–100%) containing 0.1% formic acid for 20 min followed by 99.9% methanol and 0.1% formic acid for 10 min, at a flow rate of 0.5 ml/min using *m/z* = 367.3 [M + H-H_2_O]^+^. For 20(OH)L3, 22(OH)L3, 20,22(OH)L3 and pL, an Agilent Zorbax Eclipse Plus C18 column (2.1 × 50 mm, 1.8 µm) was used with a gradient of methanol in water containing 0.1% formic acid (20–60% for 3 min then 60–100% for 1 min), followed by isocratic 99.9% methanol plus 0.1% formic acid for 2.1 min, all at at flow rate of 0.3 ml/min using *m/z* = 383.3 [M + H-H_2_O]^+^ for 20(OH)L3 and 22(OH)L3, and *m/z* = 399.3 [M + H-H_2_O]^+^ for 20,22(OH)_2_L3, and *m/z* = 297.2 [M + H-H_2_O]^+^ for pL. MS analyses were done as described before with the concentrations of metabolites being calculated from standards curves constructed using the corresponding standards^19^.

### Cell Culture

Normal human epidermal keratinocytes (NHEK) and melanocytes (NHEM) were grown in either keratinocyte media (Lonza Walkersville Inc., Walkersville, MD) or in melanocyte growth media (MGM) supplemented with either KGF or MGF (Lonza), respectively, while dermal fibroblasts were cultured in DMEM medium containing antibiotics and 10% charcoal-treated fetal bovine serum (ctFBS) as previously detailed^55, 56, 67, 68^. Cells in the third passage were used for experiments. HaCaT immortalized keratinocytes were cultured in DMEM plus 5 or 10% FBS, while SKMel-188 melanoma cells were grown in Ham’s F10 and 5 or 10% FBS as described before^55, 68^. For experimental treatments, ctFBS was used as indicated.

### Measurement of antiproliferative activity

Cells were suspended in cell-type defined media at a concentration of 1,000 cells per well in 96 well-plates. Proliferation was estimated using (3-(4,5-dimethylthiazol-2-yl)-5-(3-carboxymethoxyphenyl)-2-(4-sulfophenyl)-2H-tetrazolium) (MTS) or sulforhodamine B (SRB solutions (Promega, Madison, WI, USA) according to the manufacturer’s instructions, as described previously^55, 56, 68^, with cell-type specific details listed in the legends. Anchorage independent growth of melanoma cells was measured by their ability to grow in soft agar as detailed in refs 54 and 55.

### Real-time polymerase chain reaction (qPCR)

Briefly, total RNA was isolated from cultured normal keratinocytes and reverse transcribed into cDNA. qPCR data were generated as detailed previously^45^ and described in the table legend.

### Immunofluorescence *in situ* studies

Protein expression was measured by immunofluorescence (IF) following protocols previously described^45^. Briefly, HEM plated onto 96-well plates (see above) were further treated with 20(OH)L3 or ethanol vehicle (control) for 24 h, and then cells were fixed and prepared for immunostaining, as previously described^45^ and detailed in supplemental Figure 5.

### NFkB luciferase assay

A NFkB luciferase stable reporter cell line (NIH/3T3) from Signosis, Inc. Santa Clara, CA was used to measure changes in NFkB activity. Experimental details are in supplemental Figure 8.

### Activity on nuclear receptors

#### RORα coactivator assay

The LanthaScreen TR-FRET RORα Coactivator kit (Thermo Fisher Scientific, Inc., Waltham, MA) was used to measure the RORα coactivator activity following the manufacturer’s protocol and as detailed in figure legends.

### RORE-LUC reporter gene on CHO Tet-on cells

Doxycycline-inducible RORα or RORγ stable CHO Tet-on cells that contain the Luc reporter, pGL4–27–5xRORE, that have been previously described^24^, were used. The details of the assay are also listed in the legend to Fig. 6.

### RORE-dependent transactivation of a LUC reporter in skin cells

HaCaT keratinocytes or SKMEL-188 melanoma cells were grown in 96 well plates in DMEM containing 5% ctFBS. After 80% confluence was attained, the cells were transfected with the reporter plasmid pGL4.27-(RORE)_5_, and RORE-LUC activity was measured as previously described^24^ and in the legend to Fig. 5.

### Translocation of VDR-GFP from the cytoplasm to nucleus

SKMEL-188 cells transduced by pLenti-CMV-VDR-EGFP-pgk-puro to express a VDR-EGFP fusion protein were grown on 96 well plates and the VDR translocation assay performed as previously described^2, 62^ and in the legend to supplemental Figure 11.

### Computational methods

The crystal structures of LDDs of RORα and RORγ, and the genomic and nongenomic LBDs of VDR, were used for docking experiments and modeling as previously described^22, 24, 62^. Additional details are in the supplemental file.

### VDRE-luciferase reporter assay

HaCaT cells stably transduced with lentiviral VDRE luciferase were used for the experiments as detailed in supplemental Figure 12.

## Electronic supplementary material

Supplementary information
