# Supplementary material for: Characterization of a new pathway that activates lumisterol in vivo to biologically active hydroxylumisterols
Source: Sci Rep. 2017 Sep 12;7:11434. doi: 10.1038/s41598-017-10202-7 (PMC5595834; doi:10.1038/s41598-017-10202-7)
Supplement: Supplementary file 1 — Supplementary information [file 41598_2017_10202_MOESM1_ESM.pdf]

Supplementary Information

Characterization of a new pathway that activates lumisterol *in vivo* to biologically active hydroxylumisterols

Andrzej T. Slominski[1,2,3,4*], Tae-Kang Kim[1], Judith V. Hobrath[5], Zorica Janjetovic[1], Allen S.W. Oak[1], Arnold Postlethwaite[6], Zongtao Lin[7], Wei Li[7] , Yukimasa Takeda[8], Anton M. Jetten[8], Robert C. Tuckey[9]

[1]Department of Dermatology, [2]Comprehensive Cancer Center, Cancer Chemoprevention Program, [3]Nutrition Obesity Research Center, University of Alabama at Birmingham, [4]Pathology and Laboratory Medicine Service, VA Medical Center, Birmingham, AL 35249. USA
[5]Drug Discovery Unit, College of Life Sciences, University of Dundee, Dundee DD1 5EH, United Kingdom
[6]Department of Medicine, Division of Rheumatology and Connective Tissue Diseases, University of Tennessee HSC, and Memphis VA Medical Center, Memphis, TN 38163, USA
[7]Department of Pharmaceutical Sciences, University of Tennessee HSC, Memphis, TN 38163, USA
[8]Cell Biology Section, National Institute of Environmental Health Sciences, National Institutes of Health, Research Triangle Park, NC 27709, USA.
[9]School of Molecular Sciences, University of Western Australia, Pert, WA, Australia

*Corresponding author:
Andrzej T. Slominski, MD, PhD, Department of Dermatology, University of Alabama at Birmingham, Birmingham, AL 35249. USA; e-mail: aslominski@uabmc.edu; phone: 205.934.5245

Supplemental Figure 1. Concentrations of lumisterol (L3) and its precursor 7-dehydrocholesterol (7DHC) in the epidermis (A) in relation to race, gender and age of the donor and in the serum (B) in relation to gender and age. 7DHC and L3 were analyzed by LC-MS using a Waters Atlantis dC18 column (100 × 4.6 mm, 5 µm particle size) with a gradient of methanol in water (85–100%) containing 0.1% formic acid for 20 min followed by 100% methanol containing 0.1% formic acid for 10 min, at a flow rate of 0.5 ml/min. The concentrations of the products were calculated from MS peak areas in relation to standard curves generated using $m/z = 367.3$ $(M+H-H_2O)^+$. The individual values and the means are presented. Data were analyzed using student's t-test with $p<0.05$ considered as significant..

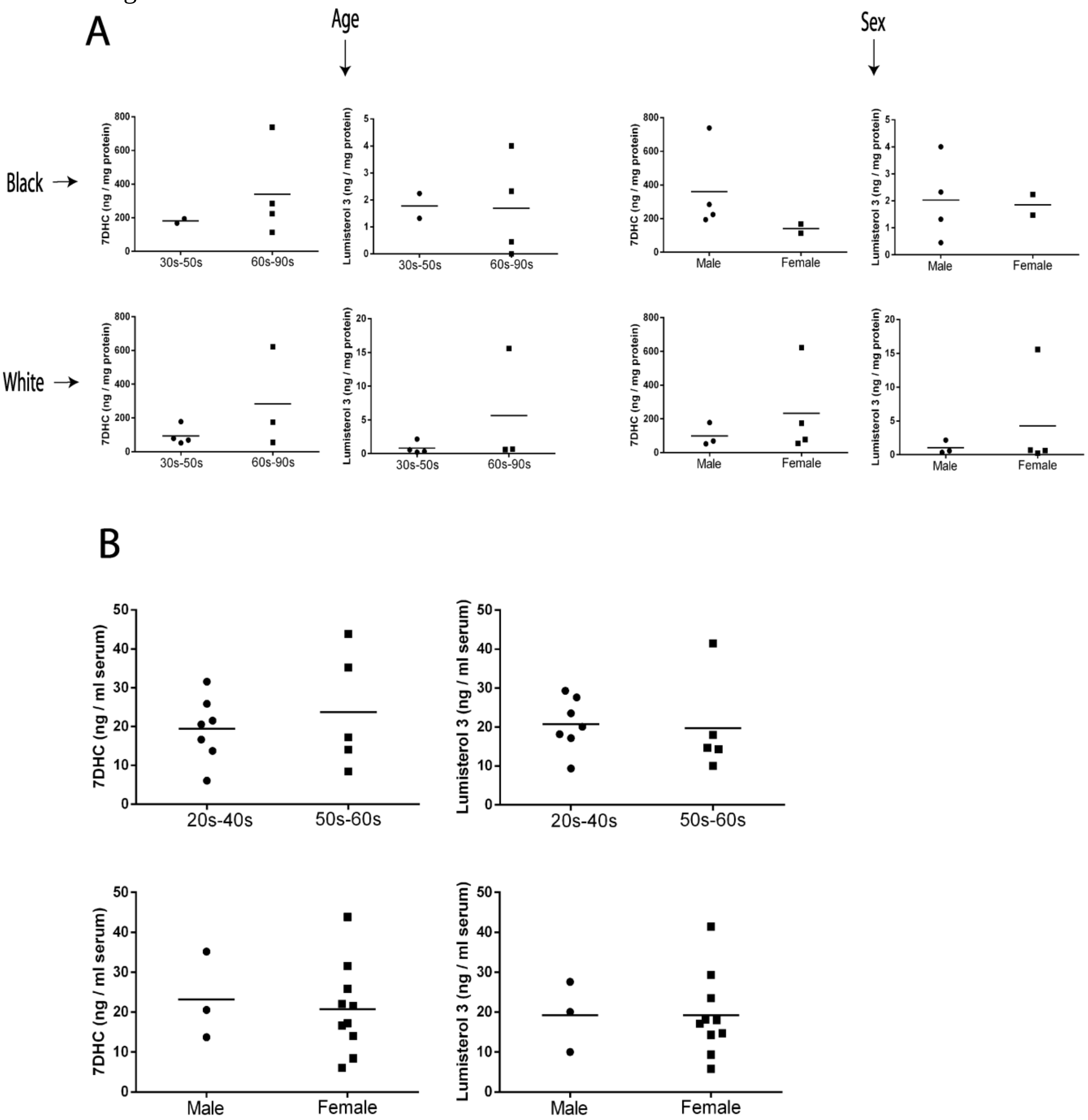

Supplemental Figure 2. Identification of 20(OH)L3 as a product of lumisterol metabolism by CYP11A1. (A) Chromatogram of products resulting from the the action of CYP11A1 on lumisterol. Lumisterol (50 mM, solubilized in 2-hydroxypropyl-β-cyclodextrin at a final concentration of 2.25%) was incubated with bovine CYP11A1 (2.5 µM) for 3 h and products extracted with dichloromethane, as before [1]. HPLC was carried out on a Grace Alltima C18 column, 0.46 x 25 cm, with a methanol in water gradient (64% to 100% methanol for 15 min then 100% methanol for 45 min, at 0.5 ml/min). Major products identified previously by NMR [1] are labelled as well as product G, previously shown to be a mono-hydroxylumisterol by mass spectrometry. (B) Standard 20(OH)L3 was chromatographed under identical conditions to panel A and gave the same retention (RT) time as product G. (C) Product G from panel A was collected and rechromatographed on the same column using an acetonitrile in water gradient (45% to 100% acetonitrile for 15 min then 100% acetonitrile for 45 min, at 0.5 ml/min. (D) Standard 20(OH)L3 was chromatographed under identical conditions to panel C and again gave the same retention time as product G. Lumisterol products were detected with a UV monitor at 280 nm.

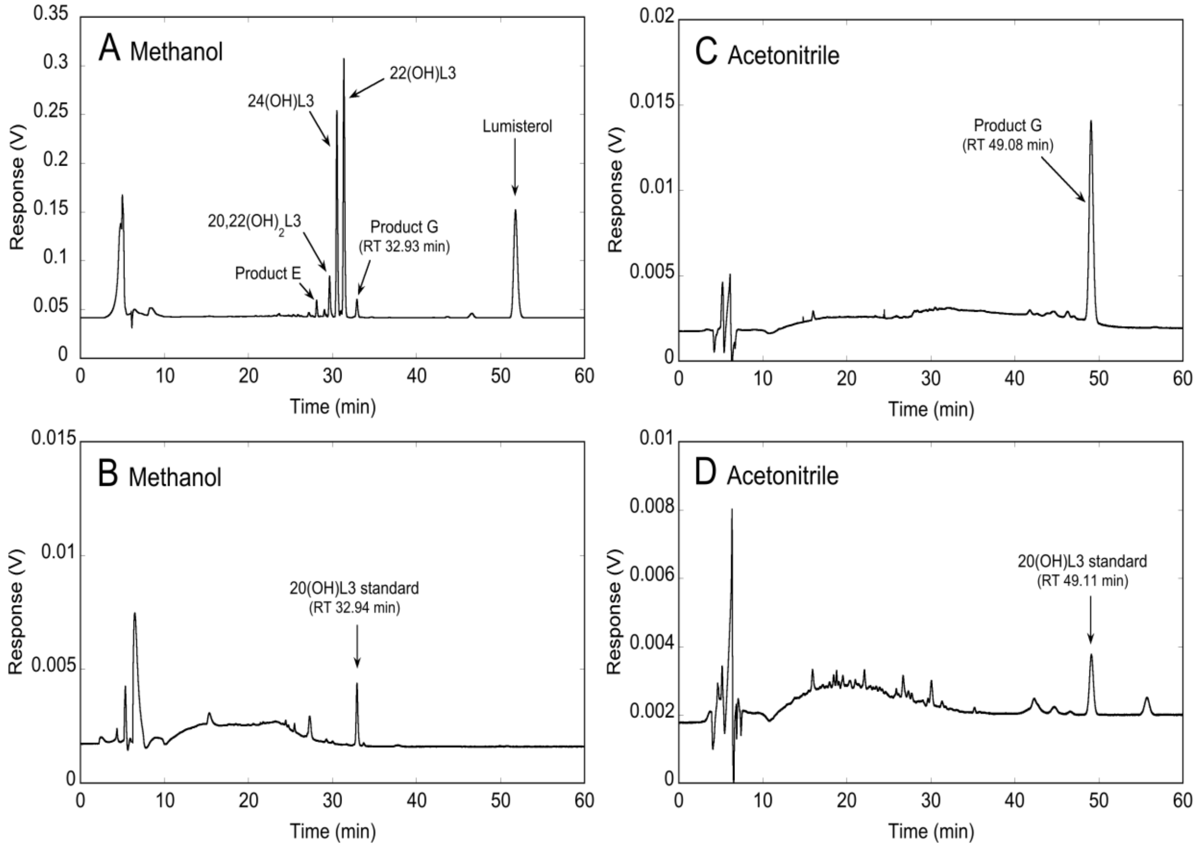

Supplemental Figure 3. 20(OH)L3 production was increased by exogenous addition of L3 to pig adrenal gland fragments (A and B) and HaCaT keratinocytes (C and D). Extracted ion chromatograms (EIC) on QTof LC-MS are shown using *m/z* = 383.3 $[M+H-H_2O]^+$. Pig adrenal gland or HaCaT keratinocytes were incubated with or without L3 for 18 h and then extracted for LC/MS analyses following protocols described previously [2-4]. A and C, no substrate; B, 500 uM L3; D, 50 uM L3. The extraction and LC-MS conditions are described in materials and methods.

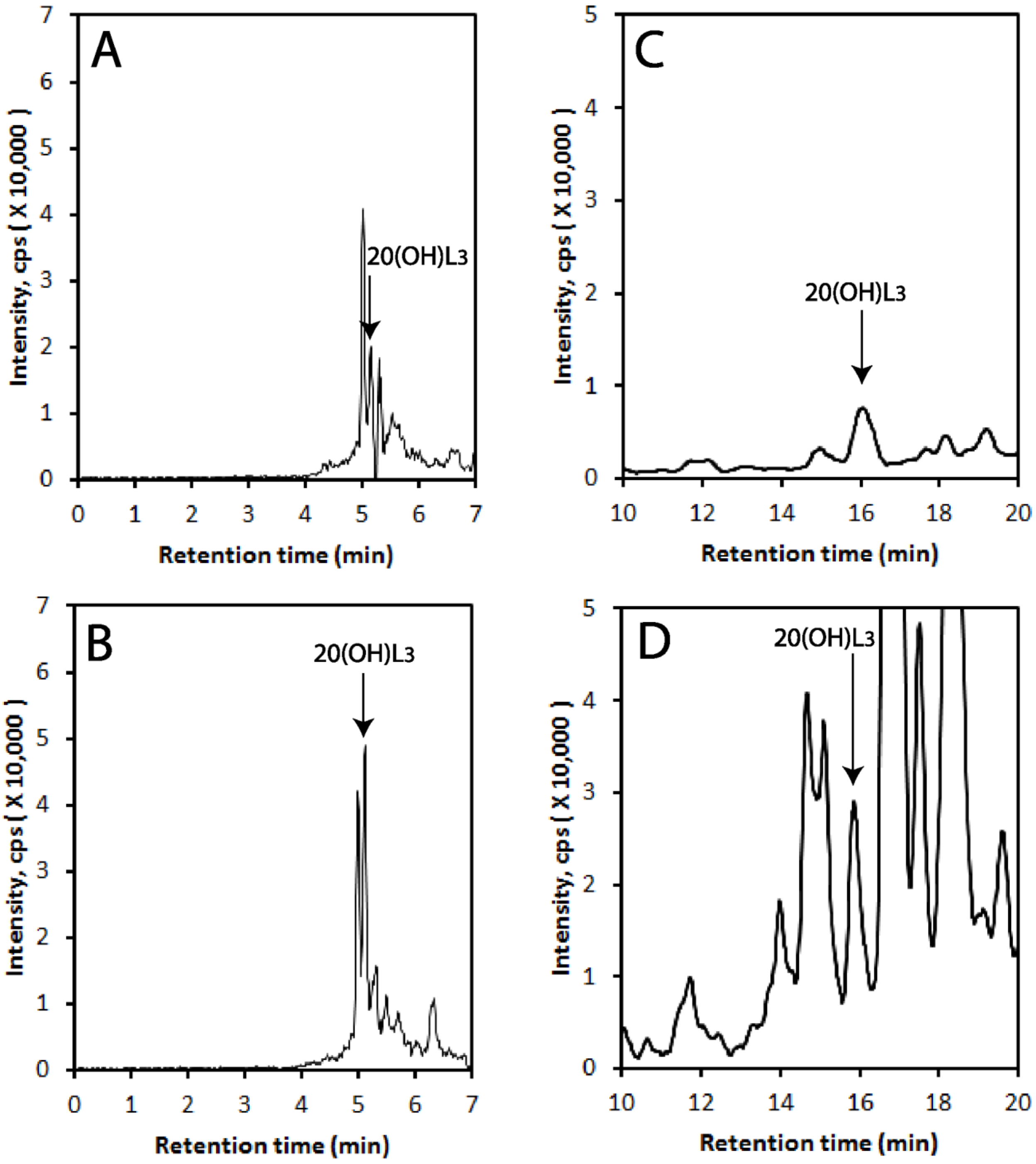

Supplemental Figure 4. Incubation of pig adrenal gland fragments with exogenously added L3 stimulates 17(OH)pL but not 17,20$(OH)_2$pL production. Extracted ion chromatograms (EIC) on QTof LC-MS are shown using *m/z* = 313.2 $[M+H-H_2O]^+$ for 17(OH)pL; 315.2 $[M+H-H_2O]^+$ for 17,20$(OH)_2$pL. Pig adrenal gland fragments were incubated with or without L3 for 18 h following protocols described previously [2-4]. A, no substrate; B, 500 uM L3. The extraction and LC-MS conditions are described in materials and methods.

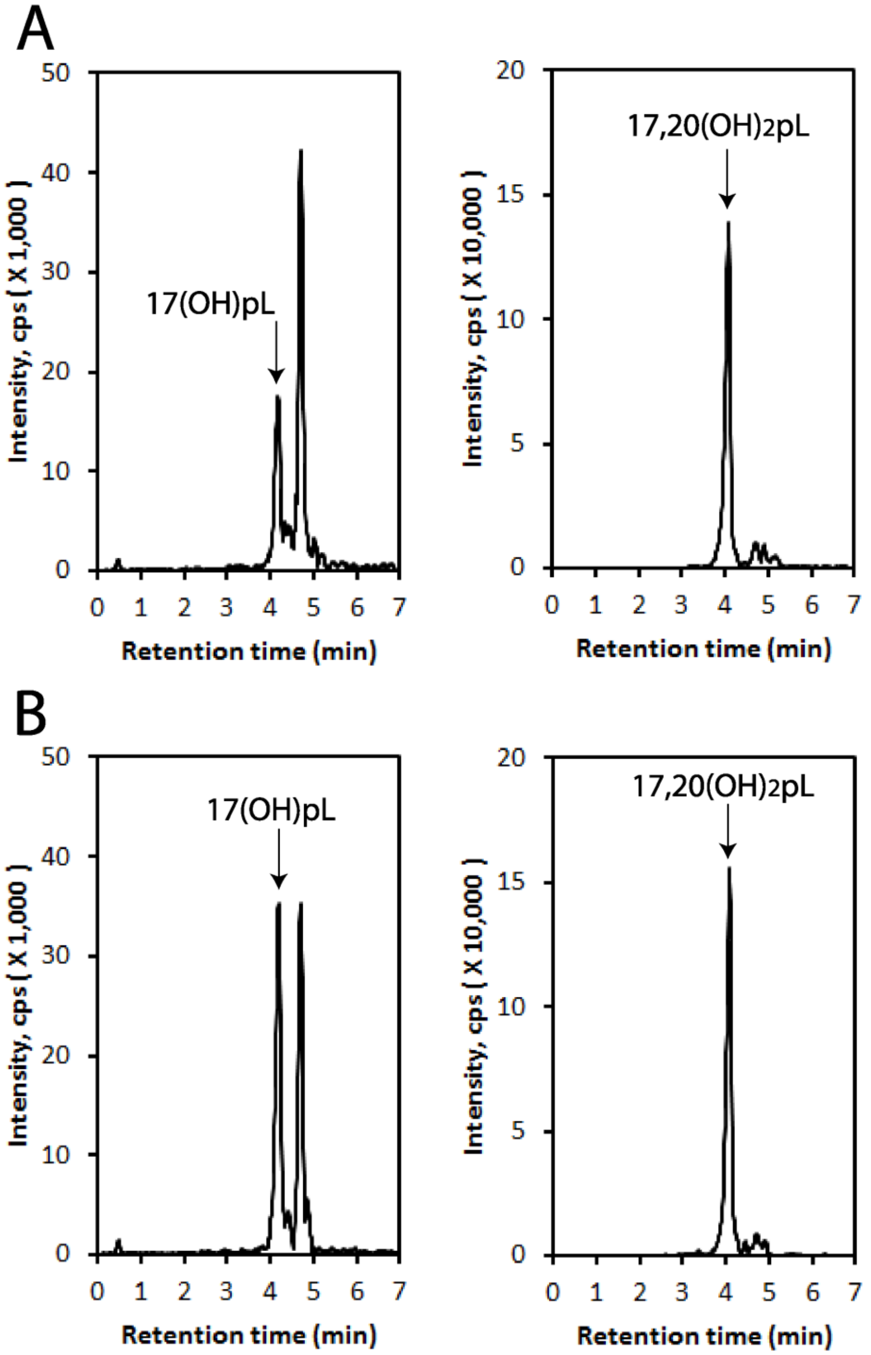

Supplemental Figure 5. 20(OH)L3 stimulates expression of involucrin and Mn-SOD. HEK cells were treated with 1 or 10 nm of 20(OH)L3 or vehicle for 24 h. Cells were fixed in 4 % paraformaldehyde and prepared for staining, as previously described [5]. Cells were stained with the following primary: Involucrin (Gene Tex (1:100) or Mn-SOD (Merck) (1:100); and secondary antibodies: anti-mouse or anti- rabbit (1:100) Alexa-Fluor 488 (Thermo-Fisher). DAPI was used to stain cell nuclei. Cells were washed five times in RT 1xPBS and scanned in PBS using Biotek Cytation 5. The fluorescent signal was detected using a Bio-Tek instrument and data were analyzed using Gen5 3.09 software. The protein expression was calculated based on the mean cellular green signal and data are presented in a form of graph created using GraphPadPrizm. Data are presented as means ± SD (n=6). Statistical significance is denoted as * p<0.05, ** P<0.01. Representative pictures are shown (vehicle, 20(OH)L3 at 1 nM or 100 nM concentrations).

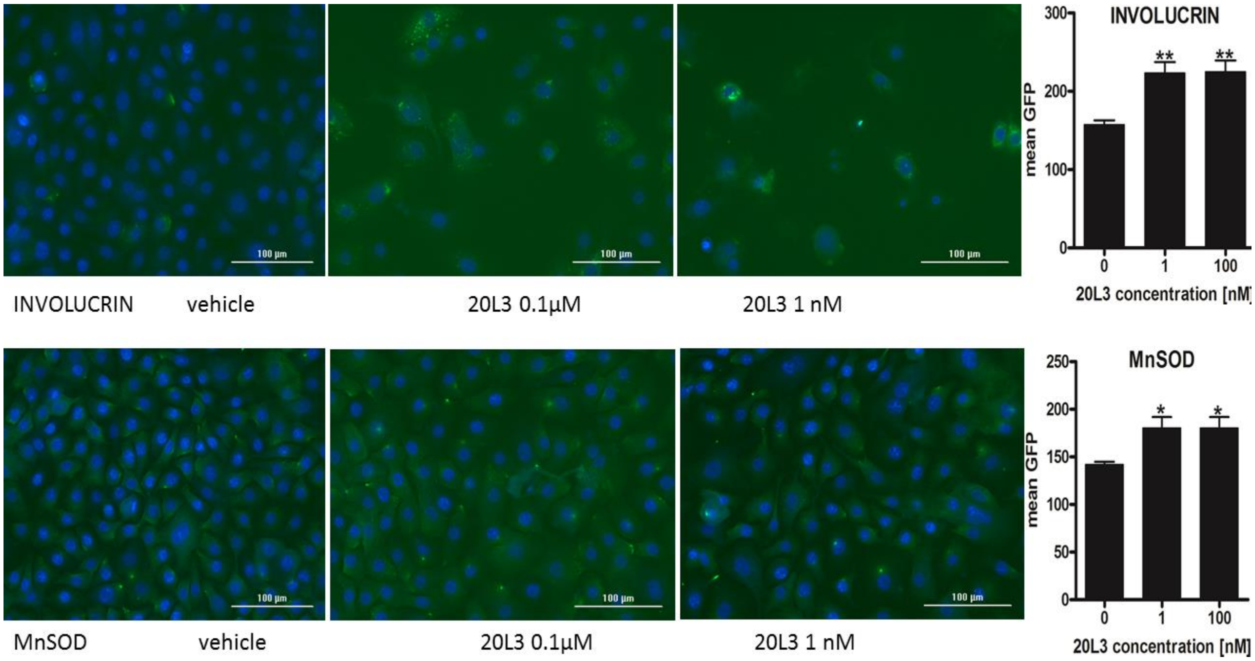

Supplemental Figure 6. Growth inhibition of normal human epidermal melanocytes by 20(OH)L3, 22(OH)L3 and 20,22$(OH)_2$L3. The cells were grown using MBM-4 media supplemented with MGM-4 (Lonza, Allendale, NJ) on 96 well plate until 30% confluence. The media were replaced with serum-free media in order to synchronize the cells for the next 24 h. Subsequently, the cells were incubated for 48 or 72 h with graded concentrations of 20(OH)L3, 22(OH)L3, 24(OH)L3, or 20,22$(OH)_2$L3 (or ethanol vehicle as a control) in MBM-4 media supplemented with MGM-4. Twenty µl of the MTS solution was added to the cultures and incubated for 3.5 h at 37°C. A Cytation 5 Cell Imaging Multi-Mode Reader (BioTek, Instruments, Inc., Winooski, VT, USA) was used to record the absorbance at 490 nm. Data represent means ± SE ($n \geq 3$) where #$p<0.05$, ##$p<0.01$ and ###$p<0.001$ by one way ANOVA test.

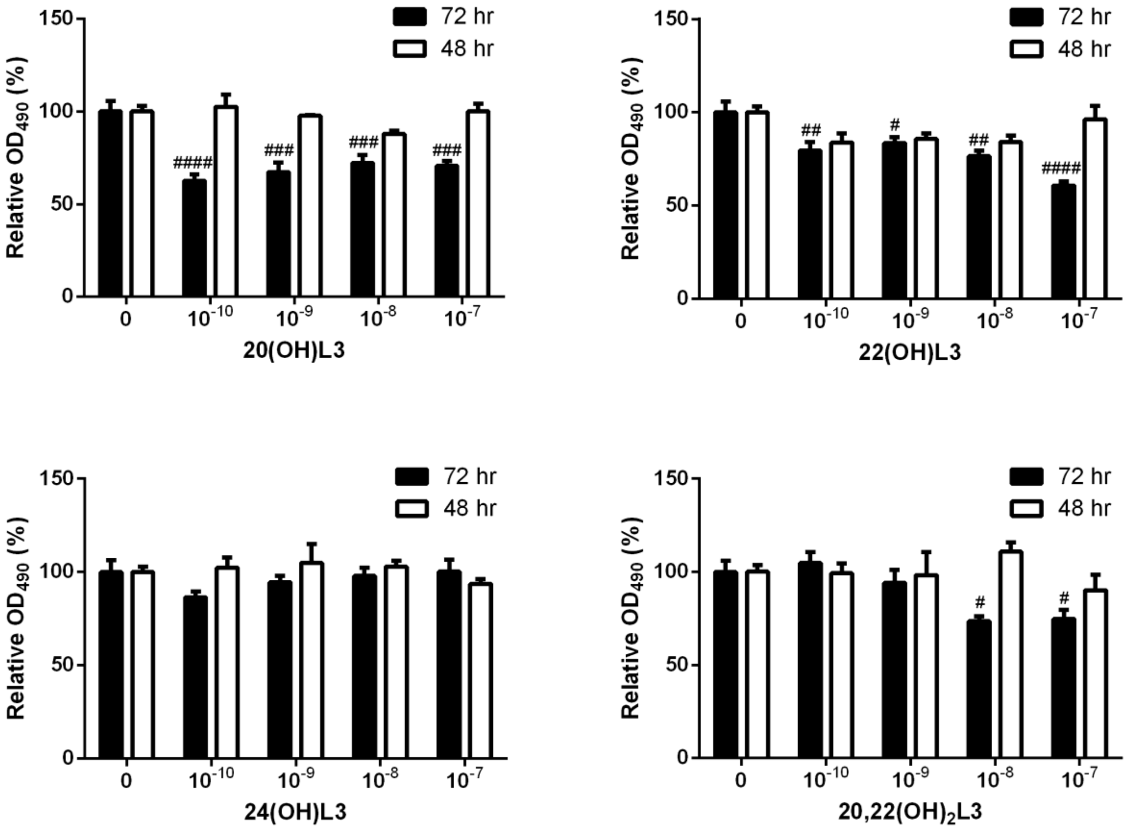

Supplemental Figure 7. Growth inhibition of normal human dermal fibroblasts by 20(OH)L3 and 22(OH)L3. The cells were grown in DMEM media containing 10% charcoal treated FBS to reach 40% confluence (for 48 h treatment) or 10% confluence (for 72 h treatment) on 96 well plates. Then the media were replaced with serum-free media in order to synchronize the cells for the next 24 h Subsequently, the cells were incubated for 48 or 72 h with graded concentrations of 20(OH)L3, 22(OH)L3, 24(OH)L3, or 20,22(OH)L3 (or ethanol vehicle as a control) in DMEM plus 10%cFBS. Cell growth was measured using MTS assay as described in legend to supplemental Figure 5. Data represent means ± SE (n ≥ 3) where #p<0.05, ##p<0.01 and ###p<0.001 at one way ANOVA test.

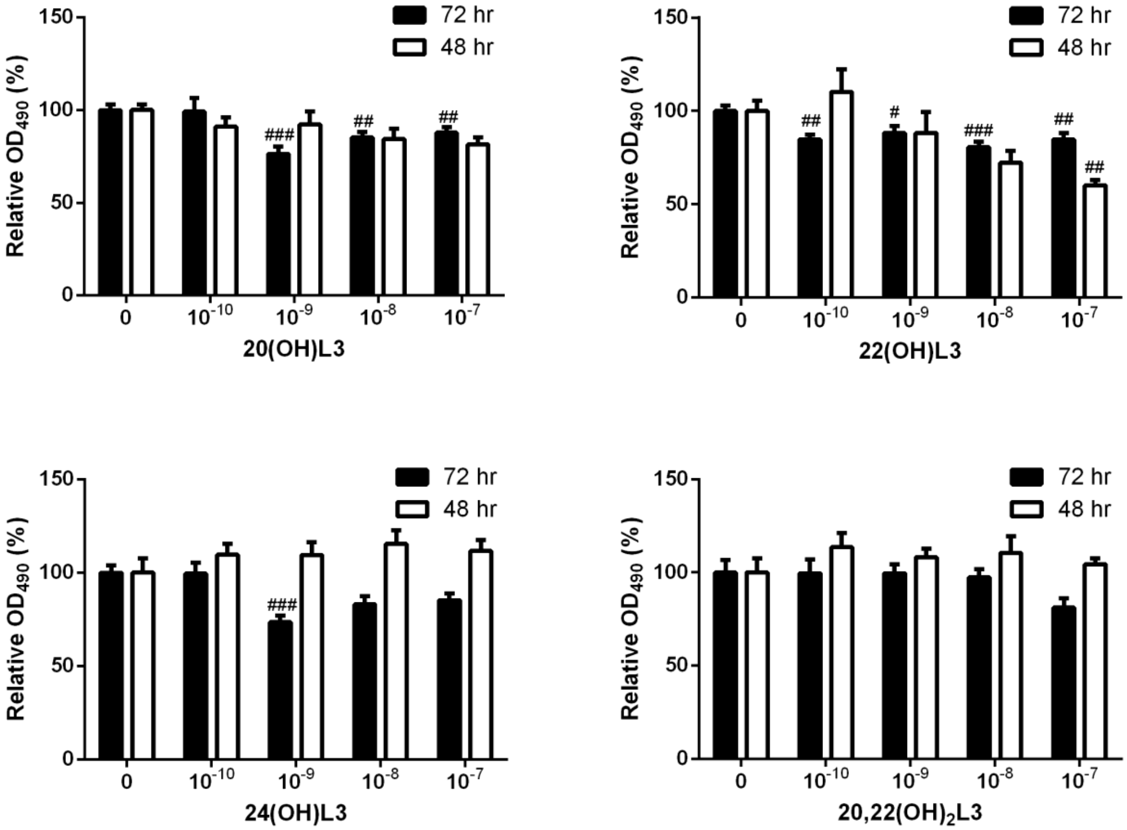

Supplemental Figure 8. Effects of hydroxylumisterols on NFkB luciferase expression in the NIH/3T3 cell line expressing the NFkB luciferase reporter gene. The cell line stably expressing NFkB luciferase reporter gene was purchased from Signosis, Inc. Santa Clara, CA and the assays were performed following the manufacturer's protocol. The cells were grown on 96 well (white) plate using DMEM media containing 10% charcoal-treated FBS until 80-90% confluence followed by treatment with hydroxylumisterols for 60 min (**A**).To measure the effect on constitutive NFkB activity. The effect of hydroxylumisterols on induced NFkB activity cells pre-treated with hydroxylumisterols for 30 (**B** and **C**) or 60 (**D**) min, then 0.25ug/ml TNFα (Sigma) was added for an additional 1 (B, D) or 2 h (C). A luciferase assay system (Promega, Madison, WI) was used for luciferase assay following the manufacturer's protocol and the relative luminescence strength was read with a Cytation 5 Cell Imaging Multi-Mode Reader (BioTek, Instruments, Inc., Winooski, VT, USA). Data represent means ± SE ($n \geq 3$) where *$p<0.05$, **$p<0.01$, ***$p<0.001$ and ****$p<0.0001$ the student t-test.

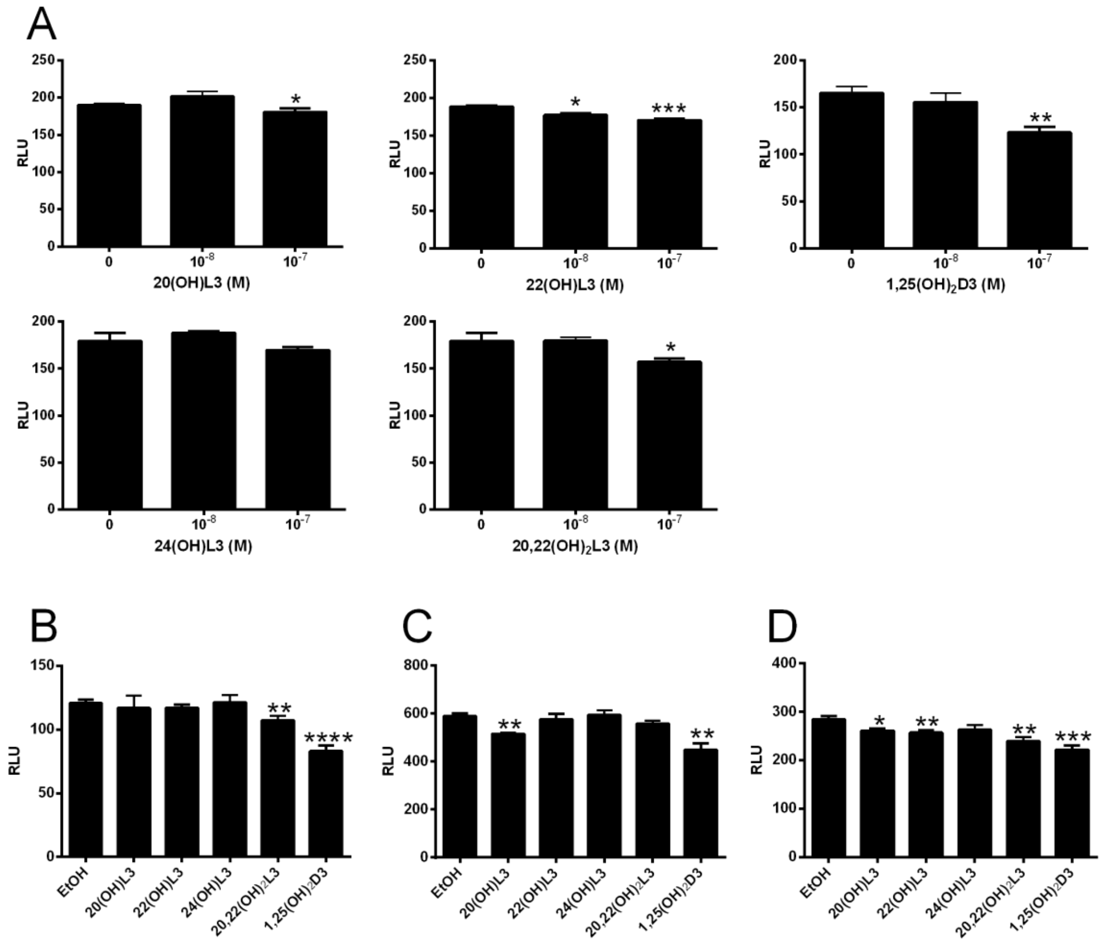

Supplemental Figure 9. Ligand-induced modifications of RORE-dependent activation of the Luc reporter in CHO Tet-on cells (**A**) and SKMEL-188 melanoma cells (**B**). **A.** Effects of 7DHC, 7DHP, 20*S*(OH)7DHC (20(OH)-7DHC-S), 20*R*(OH)7DHC (20(OH)-7DHC-R), D3, 20*S*(OH)D3 (20(OH)-D3-R), 20*R*(OH)D3 (20(OH)-D3-R), 25(OH)D3 (25(OH)-D3) and 20(OH)L3 (20(OH)-lumisterol on a 5xRORE driven LUC reporter (pGL4-27-5xRORE) by RORα and RORγ in CHO Tet-on cells were investigated as described previously [6]. Data are means±SE. **B.** Inhibition of RORE-LUC activity by 20(OH)L3 and its precursor 20(OH)7DHC in SKMEL-188 melanoma cells transfected with a reporter plasmids pGL4.27-(RORE)$_5$ [7]. Data are presented as means ±SE (*p<0.05 or **p<0.01 in t-test) and details of the assays have been described previously [6].

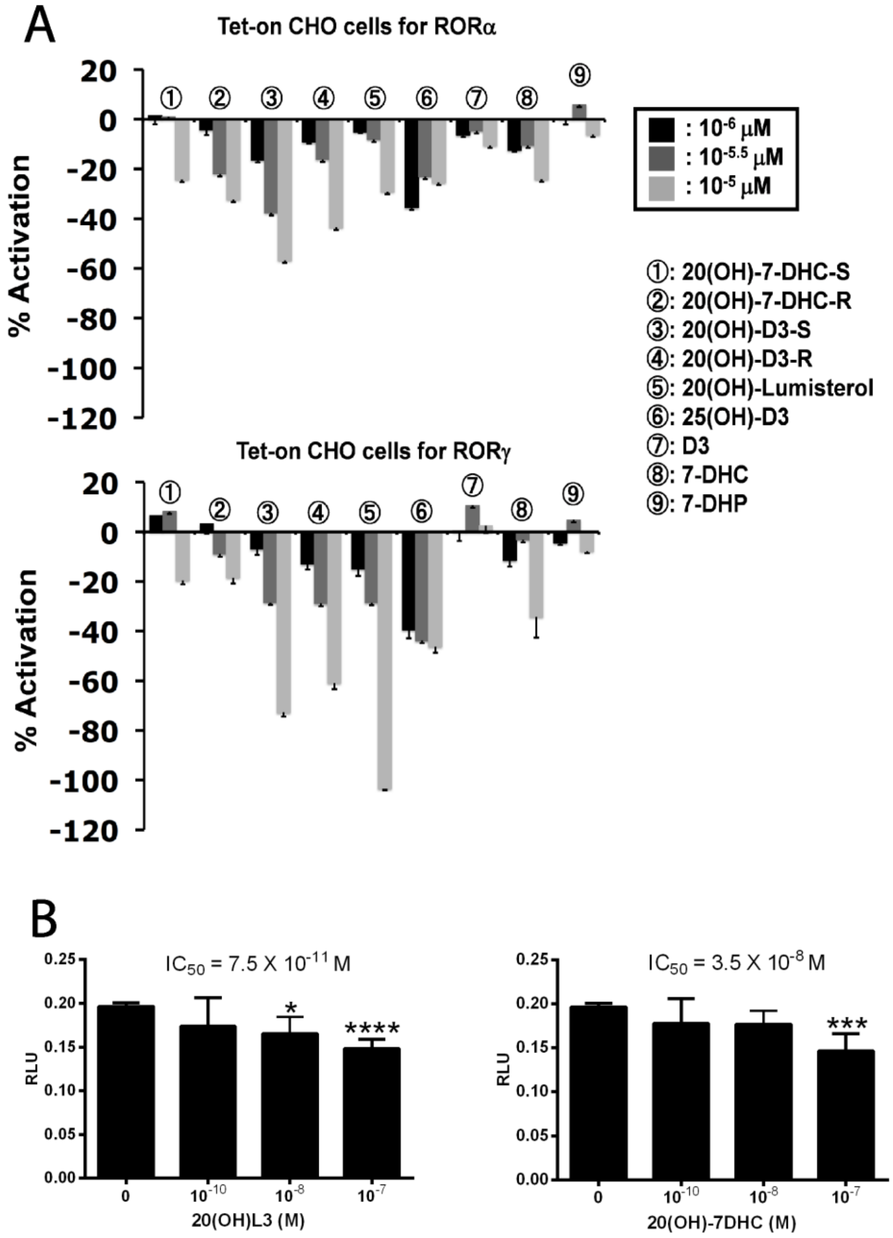

Supplemental Figure 10. Stimulation of RORα activity by 17(OH)pL, 17,20(OH)2pL and to a lesser degree by pL. The RORα coactivator assay was performed using the LanthaScreen TR-FRET RORα Coactivator kit assay. RORα-LBD was added to the concentrations of pL or 17(OH)pL or 17,20(OH)pL as indicated, followed by addition of a mixture of peptide (TRAP220/DRIP2) and antibody (Tb-anti-GST). The reaction mixture was incubated at room temperature for 2 h and TR-FRET ratio was calculated by dividing the fluorescein emission at 520 nm by the Terbium emission at 495 nm using Synergy neo2 (BioTek Instruments, Inc. Winooski, VT).

Data represent means ± SE (n > 3) where **p<0.01 and ****p<0.0001 student t-test; ##p<0.01, ###p<0.001 and ####p<0.0001 at one-way ANOVA test and general ANOVA tests are shown.

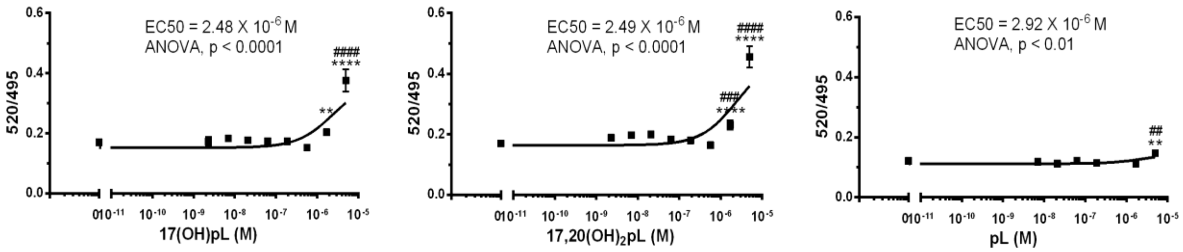

Suppemental Figure 11. 20(OH)L3 as well as monohydroxy-D3 derivatives have no effect on VDRE luciferase activity in HaCaT keratinocytes. HaCaT cells transduced with lentiviral VDRE luciferase[8,9] were used for the assay as described previously[8-11]. Briefly, HaCaT cells were seeded in 96-well plates in DMEM plus 10% cFBS (10,000 cells/100 µL/well) and incubated for 24h. Solutions of 20(OH)D3, 1,20(OH)$_2$D3, L3, 20(OH)L3, 22(OH)D3, 24(OH)D3, 20,22(OH)$_2$D3 and 1,25(OH)$_2$D3 in DMSO at a series of concentrations (10^{-4} to 10^{-7} M) were added to cells, which then underwent another 24 h incubation. The luciferase detection solution was added to measure the luciferase signal based on the manufacturer's procedure. 10% DMSO in water was used as a control, and the concentration of DMSO for cells during the treatments was 0.1%. Data are presented as mean ± SE (n = 3). The EC50 values of 1,20(OH)D3 and 1,25(OH)D3 were 323.2±18.4 and 378.7±11.5 nM, respectively.

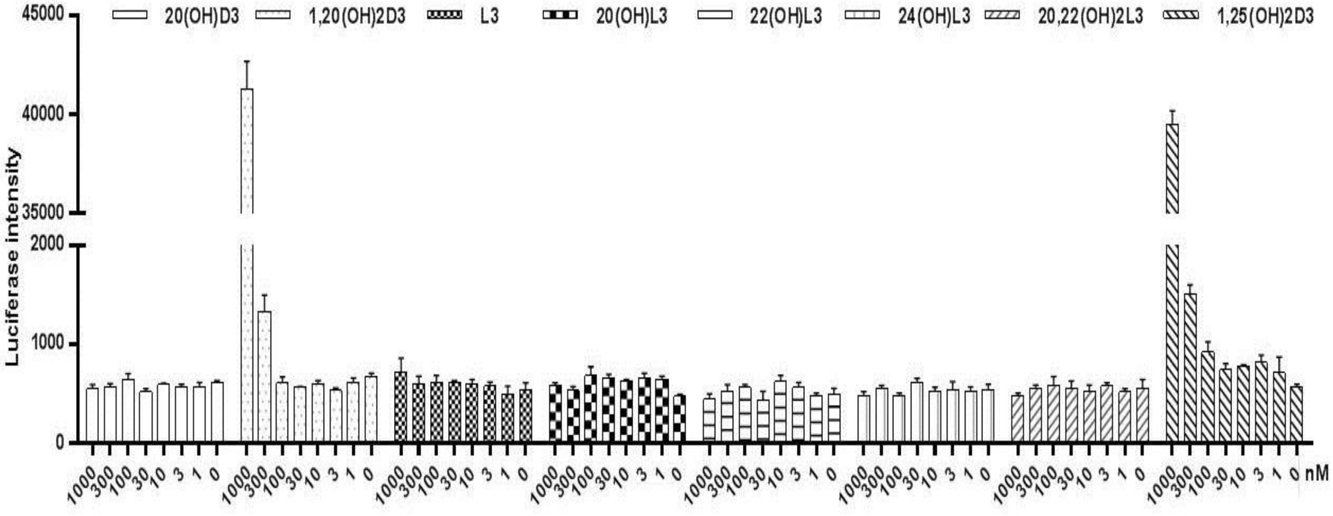

Supplemental Figure 12. Hydroxylumisterols stimulate VDR-GFP translocation from the cytoplasm to the nucleus in the SKMEL-188 melanoma line stably overexpressing VDR-GFP [12]. The cells were grown in 96 well-plates containing Ham's F10 media plus 5% charcoal-treated FBS until 50% confluence was reached. Then the cells were exposed to the graded concentration of hydroxylumisterols or vehicle (ethanol) for 12 h. The VDR translocation to the nucleus was measured as described in [13,14]. Briefly, plates were analyzed using Cytation 5 (BioTek Instruments, Inc. Winooski, VT) equipped with a digital immunofluorescence microscope and the cells expressing VDR-GFP were automatically counted from central areas of 3 different wells using 10 X magnification.
Data represent means ± SE (n=3-4). #, p<0.05; ##, p<0.01; ###, p<0.by a one-way ANOVA test.
.

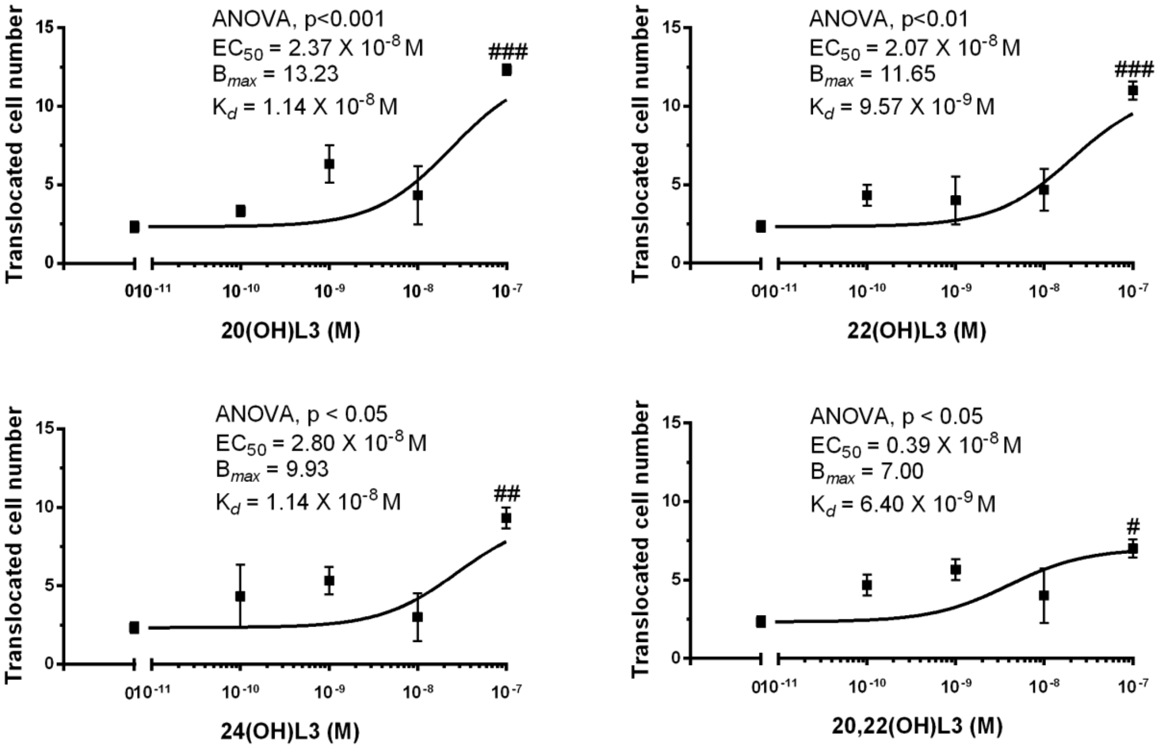

Preparation of protein structures for docking.

The following crystal structures were used for docking studies: the orphan nuclear receptor RORα co-crystallized with cholesterol at 1.63 Å resolution (PDB code 1N83), RORγ co-crystallized with 20-hydroxycholesterol at 2.35 Å resolution (PDB code 3KYT) and the vitamin D nuclear receptor (VDR) co-crystallized with 1α,25-dihydroxyvitamin D3 (1,25vitD) in the genomic site (G-pocket) at 1.80 Å resolution (PDB code 1DB1). Prior to docking all crystal structures with the exception of the structure used for docking into the VDR non-genomic site (A-pocket) were prepared as follows. Water molecules were deleted except for tightly bound crystal waters that may affect ligand binding. Structures were refined applying the Prime preparation and refinement tools of the Protein preparation wizard implemented in the Schrödinger package (version 2015-2). After addition of hydrogens and detection of disulfide bonds, protein structures (including co-crystallized ligands) were optimized through restrained minimization, applying default parameters to maximum of 0.3 root-mean-square deviation (RMSD) between refined and crystal structures. All ligand structures were prepared using the LigPrep utility of Schrödinger at pH = 7.4.

For docking into the A-pocket of VDR a ligand-induced conformation of the site was generated using 1α,25-dihydroxylumisterol as the bound ligand as follows. Since in the VDR crystal structure Tyr295 penetrates deep into the A-pocket while hydrogen bonding with the side chain of His229 both residues were mutated to Ala. The side chain conformation of Arg158 was changed to a rotamer that places the guanidinium moiety closer to Asp292. The co-crystallized ligand and all water molecules were removed except for two coordinated crystal waters in the A-pocket (HOH501 and HOH506). The structure was then refined using the Protein preparation wizard module of Schrödinger, applying restrained minimization to RMSD 0.3 (as described above). 1α,25-dihydroxylumisterol was docked into this A-pocket structure using the Induced Fit docking method in extra-precision (XP) mode (Schrödinger software) which allows for protein flexibility within 5 Å of the ligand. A favorable Induced Fit pose was selected and copied/ merged with the VDR crystal structure (PDB code 1DB1) while the protein structure from the Induced Fit run was discarded. In the crystal structure the side chain conformation of Arg158 was changed to a rotamer closer to Asp292, as done previously. The side chain rotamer of Tyr295 was adjusted and the histidine state of His229 changed (flipped) to allow Tyr295 to hydrogen bond with His229 in a 'reverse' hydrogen bonding interaction compared to the crystal structure. The obtained complex (including Induced Fit pose of the ligand) was refined using restrained minimization in Protein preparation wizard as described above. The structure was then relaxed using MacroModel suite of tools, allowing the ligand and residues within 3 Å from the ligand to move freely. Three constrained shell regions were defined that surround freely moving atoms (including the ligand). Each shell contains residues within 5 Å of the previous shell, with force constants 50 and 200 (kJ • mol-1 • Å-2) for the first two shells, respectively, and frozen atoms in the outmost shell. Using OPLS-2005 force field conjugate minimization was applied in water solvation model and extended non-bonded interactions cutoff. The final structure was further refined through restrained minimization using the Protein preparation wizard module. The obtained structure was used for all docking runs into the A-pocket of VDR and the ligand 1α,25-dihydroxylumisterol used for defining the binding site for docking.

Docking protocol.

All docking runs utilized the Glide method in Extra Precision (XP) mode as implemented in Schrödinger (Small Molecule Drug Discovery Suite 2015-2 or 2016-1). Default parameters were used (except for increasing the number of poses kept for the initial phase of docking to 7500, and

poses selected for energy minimization to 1200). No constraints were defined. Prior to docking ligand structures were prepared using the LigPrep utility of Schrödinger at pH 7.4.

Supplemental Figure 13A. Schematic representation of the interactions of lumisterol analogs in the non-genomic site (A-pocket). Residues contributing to favorable contacts are colored coded as follows: red for polar, brown for non-polar, black for residues involved in both polar/non-polar interactions. Hydrogen bonds are shown with dashed lines; '-b' indicates backbone atoms.

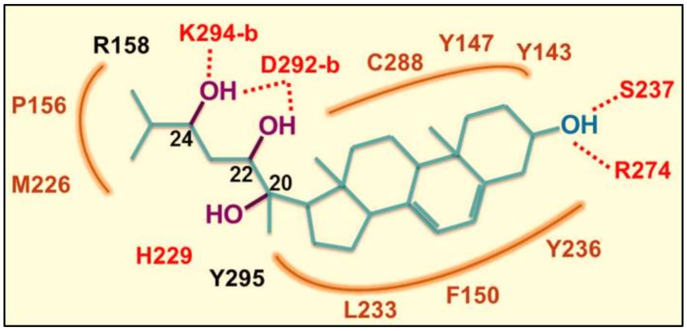

Supplemental Figure 13B. Docked pose of 1α,25-dihydroxylumisterol in the A-pocket of VDR. Ligand carbons are colored orange (all other atoms by atom type as in Fig. 7). Hydrogen bonding interactions are indicated with dashed lines. Only residues contributing to ligand binding are shown.

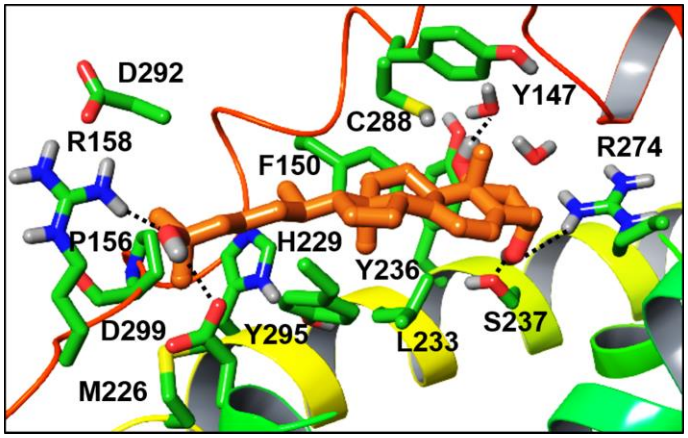

Supplemental Figure 14. Representative 22 or 24-substituted hydroxylumisterol analogs docked into the non-genomic site of VDR. Ligand carbons are color coded as shown (all other atoms are colored by atom type as in Fig.7). Hydrogen bonding interactions are indicated with dashed lines. A. 20,22-hydroxylumisterol and B. 24-hydroxylumisterol.

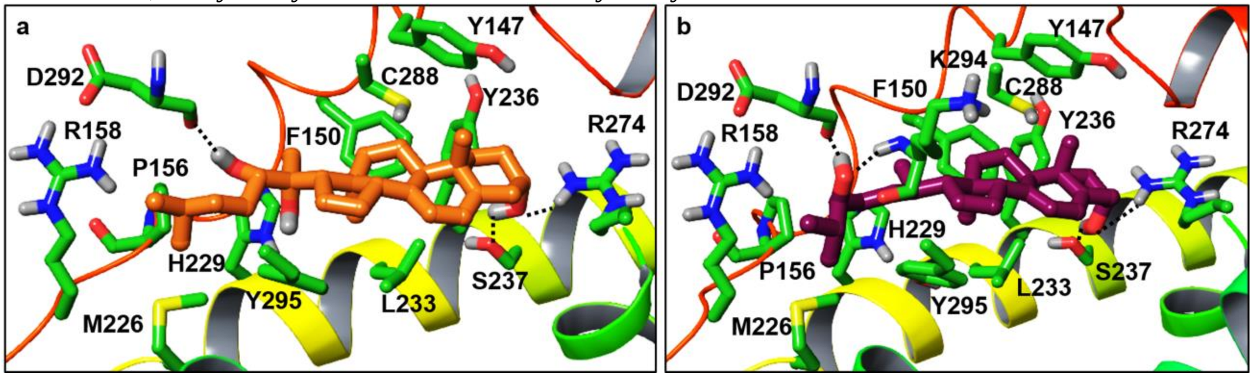

Results for docking to the VDR.

Tyr295 penetrates deep into the non-genomic site (A-pocket) in the VDR crystal structure (PDB code 1DB1) interfering with modelling ligands into the site. As described under 'Preparation of protein structures for docking', prior to docking an induced conformation was generated for the A-pocket region that is biased toward the binding of 1,25$(OH)_2$L3. The series of lumisterol analogs were docked into the induced A-pocket structure of VDR using Glide in extra-precision mode (XP), as implemented in the Schrödinger package (version 2015-2). Obtained poses show favorable binding and Glide XP scores as listed in Table 3. Since poses of all lumisterol analogs closely overlap, interactions formed by analogs within this series may be represented schematically as illustrated in Supplemental Fig. 13A. Non-polar contacts are similar to those predicted for 1,25$(OH)_2$L3 (Supplemental Fig. 13B). Common to all lumisterol analogs is a near optimal hydrogen bonding interaction between the 3-hydroxyl and Ser237 while this hydroxyl group also forms a hydrogen bond with the guanidinium moiety of Arg274. Hydroxyl substituents introduced at positions 22 or 24 each contribute with hydrogen bonding interactions as shown in case of two representative analogs in supplemental Fig. 14. The 20-hydroxyl group occupies a polar region near His229 and Tyr295. In conclusion, docking results suggest good fit and favorable binding in the A-pocket of VDR. Glide XP scores of -12.30, -12.93, -13.74 and -12.72 for 20(OH)L3, 22(OH)L3, 24(OH)L3 20,22$(OH)_2$D3, respectively, are comparable or better than that for 1,25$(OH)_2$L3 (-12.82) and 1,25$(OH)_2$D3 (-12.24). For comparison, hydroxylumisterols were also docked into the genomic (G)-pocket of VDR, where these compounds show less favorable interactions and poorer docking scores than that for 1,25$(OH)_2$D3. Glide XP scores of hydroxylumisterol analogs are in the range of -9 to -10 versus -11.40 for 1,25$(OH)_2$L3 and -15.07 for 1,25$(OH)_2$D3 (Table 3). Therefore, docking results predict that the preferred binding site of hydroxylumisterols is the non-genomic site of VDR.

Supplemental table 1. Statistical analysis of the data shown in table 1 .

**Serum**	20(OH)L3	22(OH)L3	20,22$(OH)_2$L3	pL	L3	D3	7DHC
20(OH)L3	NA	***	****	****	**	***	**
22(OH)L3	c	NA	*	**	**	N/S	****
20,22$(OH)_2$L3	****	*	NA	N/S	****	N/S	****
pL	****	**	N/S	NA	****	***	****
L3	**	**	****	****	NA	****	N/S
D3	***	N/S	N/S	***	****	NA	****
7DHC	**	****	****	****	N/S	****	NA
**Epidermis**	20(OH)L3	22(OH)L3	20,22$(OH)_2$L3	pL	L3	D3	7DHC
20(OH)L3	NA	N/S	N/S	*	N/S	*	***
22(OH)L3	N/S	NA	*	*	*	*	***
20,22$(OH)_2$L3	N/S	*	NA	N/S	N/S	N/S	***
pL	*	*	N/S	NA	*	*	***
L3	N/S	*	N/S	*	NA	****	N/S
D3	*	*	N/S	*	****	NA	***
7DHC	***	***	***	***	N/S	***	NA

The statistical analysis was performed using student t-test **,p<0.01; ***,p<0.001; ****,p<0.0001; N/S, no significant; NA, not applicable

Supplemental table 2. Sequences of primers used in table 2. Additional details are in [5,6,15-18]

Gene	Description	Sequence (l and r)
ALDOA	Aldolase A	CGGGAAGAAGGAGAACCTG<br>GACCGCTCGGAGTGTACTTT
BCL2	B-cell lymphoma 2	AGTACCTGAACCGGCACCT<br>GGCCGTACAGTTCCACAAA
BNIP3	BCL2 adenovirus E1B interacting protein	CCGGGATGCAGGAGGAGAG<br>TTATAAATAGAAACCGAGGCTGGAAC
CAT	Catalase	CGTGCTGAATGAGGAACAGA<br>AGTCAGGGTGGACCTCAGTG
CD14	(cluster of differentiation 14)	GTTCGGAAGACTTATCGACCAT<br>ACAAGGTTCTGGCGTGGT
KRT10	Cytokeratin 10	GGCTCTGGAAGAATCAAACTATGAGC<br>GGATGTTGGCATTATCAGTTGTTAGG
KRT1	Cytokeratin 1	GTTCCAGCGTGAGGTTTGTT<br>TAAGGCTGGGACAAATCGAC
KRT5	Cytokeratin 5	CTGTCTCCCGCACCAGCTTCACCTCC<br>CTCCACAAGCACCCGCAAGGCTGACC
CRH	Corticotropin releasing hormone	CTCCGGGAAGTCTTGGAAAT<br>GTTGCTGTGAGCTTGCTGTG
CYPB/PPIB	Cyclophilin B, Peptidyl-prolyl cis-trans isomerase B	TGTGGTGTTTGGCAAAGTTC<br>GTTTATCCCGGCTGTCTGTC

CYP1B1	Cytochrome P450, family1, subfamily B, polypeptide 1	CACTGCCAACACCTCTGTCTT<br>CAAGGAGCTCCATGGACTCT
CYP11A1	Cytochrome P450, family 11, subfamily A, polypeptide 1, exon 4	CCAGACCTGTTCCGTCTGTT<br>AAAATCACGTCCCATGCAG
CYP11B1	Cytochrome P450, family 11, subfamily B, polypeptide 1	AGGTGGACAGCCTGCATC<br>CCATTCAGGCCCATTCAG
CYP17A1	Cytochrome P450, family 17, subfamily A, polypeptide 1	GCATCATAGACAACCTGAGCAA<br>GGGTTTTGTTGGGGAAAATC
CYP21A2	Cytochrome P450, family 21, subfamily A, polypeptide 2, variant 2	GCTTGGCCTGACTCAGAAAT<br>GACACCAGCTTGTCTTGCAG
DHCR7	Sterol Δ7-reductase	TTGTAAAAGAAATTGCCTGTGAAT<br>GCCATGGTCAAGGGCTAC
EGFR	Epidermal growth factor receptor	ACACAGAATCTATACCCACCAGAGT<br>ATCAACTCCCAAACGGTCAC
FGF23	Fibroblast growth factor 23	CAGCATGAGCGTCCTCAGAG<br>GCCAGCATCCTCTGATCTGATC
FLG	Filaggrin	GGCACTCATCATGCAGAGAA<br>ATGGTGTCCTGACCCTCTTG
GCS	Gluthamylcysteine synthetase	TTGCAGGAAGGCATTGATCA<br>GCATCATCCAGGTGTATTTTCTCTT
GSN	Gelsolin	GCCCATGATCATCTACA<br>CATGGTTGGAGTTCAGTG
SLC2A1	Glucose transporter 1 (Glut 1)	CCATTGGCTCCGGTATCGT<br>TGCTCGCTCCACCACAAAC
GPX	Glutathione peroxidase	ACGATGTTGCCTGGAACTTT<br>GATGTCAGGCTCGATGTCAA
GSR	Glutathione reductase	GAGATGGCAGGGATCCTGTCAGC<br>GAGATGGCAGGGATCCTGTCAGC
GSTP1	Gluthatione S transferase	CCTGGTGGACATGGTGAATGA<br>CCTGGTGCAGATGCTCACATAGT
HIF1A	Hypoxia inducible factor α	TTTTTCAAGCAGTAGGAATTGGA<br>GTGATGTAGTAGCTGCATGATCG
HK2	Hexokinase	CAAAGTGACAGTGGGTGTGG<br>GCCAGGTCCTTCACTGTCTC
HSD3B1	3β-hydroxysteroid dehydrogenase/Δ-5-4 isomerase	CTTGGACAAGGCCTTCAGAC<br>TCAAGTACAGTCAGCTTGGTCCT

HSD11B1	Hydroxysteroid (11-beta) dehydrogenase 1	CAATGGAAGCATTGTTGTCG<br>GGCAGCAACCATTGGATAAG
HSD11B2	Hydroxysteroid (11-beta) dehydrogenase 2	GTCAAGGTCAGCATCATCCA<br>CACTGACCCACGTTTCTCAC
ICAM	Intracellular adhesion molecule	CCTTCCTCACCGTGTACTGG<br>AGCGTAGGGTAAGGTTCTTGC
IL1A	Interleukin 1 alpha	GGTTGAGTTTAAGCCAATCCA<br>TGCTGACCTAGGCTTGATGA
IL1B	Interleukin 1 beta	CRGTCCTGCGTGTTGAAAGA<br>TTGGGTAATTTTTGGGATCTACA
IL2	Interleukin 3	AAGTTTTACATGCCCAAGAAGG<br>AAGTGAAAGTTTTTGCTTTGAGCTA
IL4	Interleukin 4	CACCGAGTTGACCGTAACAG<br>GCCCTGCAGAAGGTTTCC
IL5	Interleukin 5	GGTTTGTTGCAGCCAAAGAT<br>TCTTGGCCCTCATTCTCACT
IL6 (IFNβ2)	Interleukin 6	GAAGCTCTATCTCGCCTCCA<br>AGCAGGCAACACCAGGAG
CXCL8	C-X-C motif chemokine ligand 8, IL8	AGACAGCAGAGCACACAAGC<br>ATGGTTCCTTCCGGTGGT
IL10	Interleukin 10	TGGGGGAGAACCTGAAGAC<br>CCTTGCTCTTGTTTTCACAGG
IL17A	Interleukin 17A	TGGGAAGACCTCATTGGTGT<br>GGATTTCGTGGGATTGTGAT
IL 22	Interleukin	GCTTGACAAGTCCAACTTCCA<br>GCTCACTCATACTGACTCCGTG
INFA1	Interferon alpha 1	CCCTCTCTTTATCAACAAACTTGC<br>TTGTTTTCATGTTGGACCAGA
INFB1	Interferon beta 1	GATGAGTACAAAAGTCCTGATCCA<br>CTGCAGCCACTGGTTCTGT
INFG	Interferon gamma	GGCATTTTGAAGAATTGGAAAG<br>TTTGGATGCTCTGGTCATCTT
INL	Involucrin	TGCCTCAGCCTTACTGTGAGT<br>TCATTTGCTCCTGATGGGTA
LDHA	Lactate dehydrogenase A	ACCCAGTTTCCACCATGATT<br>CCCAAAATGCAAGGAACACT
LOR	Loricrin	GTGGGAGCGTCAAGTACTCC<br>AGAGTAGCCGCAGACAGAGC
NHEI	Na/H exchange inhibitor	TCTGCCGTCTCCACTGTCTCCA<br>CCCTTCAGCTCCTCATTCACCA
NR3C1	Glucocorticoid receptor	CTTCAAAAGAGCAGTGGAAGGT<br>GCATGCTGGGCAGTTTTT

PDGFA	Platelet derived growth factor	GCAGTCAGATCCACAGCATC<br>TCCAAAGAATCCTCACTCCCTA
POMC	Proopiomelanocortin	AGCCTCAGCCTGCCTGGAA<br>CAGCAGGTTGCTTTCCGTGGTG
RANTES	Chemocine (C-C motif) ligand	TGCCCACATCAAGGAGTATTT<br>TTTCGGGTGACAAAGACGA
RORA	Retinoid acid-related Orphan receptor α	GTCAGCAGCTTCTACCTGGAC<br>GTGTTGTTCTGAGAGTGAAAGGCACG
RORC	Retinoid acid-related Orphan receptor γ	CAGCGCTCCAACATCTTCT<br>CCACATCTCCCACATGGACT
SOD1	Superoxide dismutase/Cu/Zn	GGCAAAGGTGGAAATGAAGA<br>GGGCCTCAGACTACATCCAA
SOD2	Superoxide dismutase/Mn	GCTCATGCTTGAGACCCAAT<br>CACCCGATCTCGACTGATTT
TGFA	Transforming growth factor	TTGCTGCCACTCAGAAACAG<br>ATCTGCCACAGTCCACCTG
TGFB1	Transforming growth factor	GCAGCACGTGGAGCTGTA<br>CAGCCGGTTGCTGAGGTA
TGFB2	Transforming growth factor	CCAAAGGGTACAATGCCAAC<br>CAGATGCTTCTGGATTTATGGTATT
TGM1	Transglutaminase	TCTGTGGGTCCTGTCCCATCCATCCTGACC<br>CCCCAACGGCCCACATCGGAACGTGGCCCATCCATCATGC
TNFA	Tumor necrosis factor alpha	CAGCCTCTTCTCCTTCCTGAT<br>GCCAGAGGGCTGATTAGAGA
TXNRD1	Thioredoxin reductase cytosolic	GAAGATCTTCCCAAGTCCTATGAC<br>ATTTGTTGCCTTAATCCTGTGAGG
TRN	Thioredoxin	TGAAGCAGATCGAGAGCAAGAC<br>TTCATTAATGGRGGCRRCAAGC
URN	Urocortin	CGAGCAGAACCGCATCATATT<br>ACAGTGCCCTGGTGGCTCT
VEGF	Vascular endothelial growth factor	TGGAATTGGATTCGCCATTT<br>TGGGTGGGTGTGTCTACAGGA

References

1 Tuckey, R. C. *et al.* Lumisterol is metabolized by CYP11A1: discovery of a new pathway. *Int J Biochem Cell Biol* **55**, 24-34, doi:10.1016/j.biocel.2014.08.004 (2014).

2 Slominski, A. T. *et al.* In vivo production of novel vitamin D2 hydroxy-derivatives by human placentas, epidermal keratinocytes, Caco-2 colon cells and the adrenal gland. *Mol Cell Endocrinol* **383**, 181-192, doi:10.1016/j.mce.2013.12.012 (2014).

3 Slominski, A. T. *et al.* Cytochrome P450scc-dependent metabolism of 7-dehydrocholesterol in placenta and epidermal keratinocytes. *Int J Biochem Cell Biol* **44**, 2003-2018, doi:10.1016/j.biocel.2012.07.027 (2012).

4 Slominski, A. T. *et al.* In vivo evidence for a novel pathway of vitamin D(3) metabolism initiated by P450scc and modified by CYP27B1. *FASEB J* **26**, 3901-3915, doi:10.1096/fj.12-208975 (2012).

5 Janjetovic, Z., Tuckey, R. C., Nguyen, M. N., Thorpe, E. M., Jr. & Slominski, A. T. 20,23-dihydroxyvitamin D3, novel P450scc product, stimulates differentiation and inhibits proliferation and NF-kappaB activity in human keratinocytes. *J Cell Physiol* **223**, 36-48 (2010).

6 Slominski, A. T. *et al.* RORalpha and ROR gamma are expressed in human skin and serve as receptors for endogenously produced noncalcemic 20-hydroxy- and 20,23-dihydroxyvitamin D. *FASEB J* **28**, 2775-2789, doi:10.1096/fj.13-242040 (2014).

7 Takeda, Y. & Jetten, A. M. Prospero-related homeobox 1 (Prox1) functions as a novel modulator of retinoic acid-related orphan receptors alpha- and gamma-mediated transactivation. *Nucleic Acids Res* **41**, 6992-7008, doi:10.1093/nar/gkt447 (2013).

8 Lin, Z. *et al.* Chemical Synthesis and Biological Activities of 20S,24S/R-Dihydroxyvitamin D3 Epimers and Their 1alpha-Hydroxyl Derivatives. *J Med Chem* **58**, 7881-7887, doi:10.1021/acs.jmedchem.5b00881 (2015).

9 Wang, Q. *et al.* Total synthesis of biologically active 20S-hydroxyvitamin D3. *Steroids* **104**, 153-162, doi:10.1016/j.steroids.2015.09.009 (2015).

10 Lin, Z. *et al.* Synthesis and Biological Evaluation of Vitamin D3 Metabolite 20S,23S-Dihydroxyvitamin D3 and Its 23R Epimer. *J Med Chem* **59**, 5102-5108, doi:10.1021/acs.jmedchem.6b00182 (2016).

11 Lin, Z. *et al.* Design, Synthesis and Biological Activities of Novel Gemini 20S-Hydroxyvitamin D3 Analogs. *Anticancer Res* **36**, 877-886 (2016).

12 Slominski, A. T. *et al.* 20-Hydroxyvitamin D2 is a noncalcemic analog of vitamin D with potent antiproliferative and prodifferentiation activities in normal and malignant cells. *Am J Physiol Cell Physiol* **300**, C526-541, doi:10.1152/ajpcell.00203.2010 (2011).

13 Kim, T. K. *et al.* Correlation between secosteroid-induced vitamin D receptor activity in melanoma cells and computer-modeled receptor binding strength. *Mol Cell Endocrinol* **361**, 143-152, doi:10.1016/j.mce.2012.04.001 (2012).

14 Slominski, A. T. *et al.* Endogenously produced nonclassical vitamin D hydroxy-metabolites act as "biased" agonists on VDR and inverse agonists on RORalpha and RORgamma. *J Steroid Biochem Mol Biol* **DOI: 10.1016/j.jsbmb.2016.09.024**, doi:10.1016/j.jsbmb.2016.09.024 (2016).

15 Janjetovic, Z. *et al.* Melatonin and its metabolites ameliorate ultraviolet B-induced damage in human epidermal keratinocytes. *J Pineal Res* **57**, 90-102, doi:10.1111/jpi.12146 (2014).
16 Slominski, A. *et al.* The role of melanogenesis in regulation of melanoma behavior: melanogenesis leads to stimulation of HIF-1alpha expression and HIF-dependent attendant pathways. *Arch Biochem Biophys* **563**, 79-93, doi:10.1016/j.abb.2014.06.030 (2014).
17 Janjetovic, Z. *et al.* Melatonin and its metabolites protect human melanocytes against UVB-induced damage: Involvement of NRF2-mediated pathways. *Sci Rep* **7**, 1274, doi:10.1038/s41598-017-01305-2 (2017).
18 Slominski, A. T. *et al.* Products of vitamin D3 or 7-dehydrocholesterol metabolism by cytochrome P450scc show anti-leukemia effects, having low or absent calcemic activity. *PLoS One* **5**, e9907 (2010).
